# Supplementary material for: A multiethnic genome-wide association study of primary open-angle glaucoma identifies novel risk loci
Source: Nat Commun. 2018 Jun 11;9:2278. doi: 10.1038/s41467-018-04555-4 (PMC5995837; doi:10.1038/s41467-018-04555-4)
Supplement: Supplementary file 2 — Supplementary Information [file 41467_2018_4555_MOESM2_ESM.pdf]

Supplementary Information for “A multiethnic genome-wide association study of primary open-angle glaucoma identifies novel risk loci”

Choquet et al.

Supplementary Figure 1. QQ plots and genomic inflation factors (λ) observed in **a.** the discovery GERA multi-ethnic meta-analysis of POAG, and each race/ethnicity group: **b.** non-Hispanic white, **c.** Hispanic/Latino, **d.** East Asian, and **e.** African-American

a.

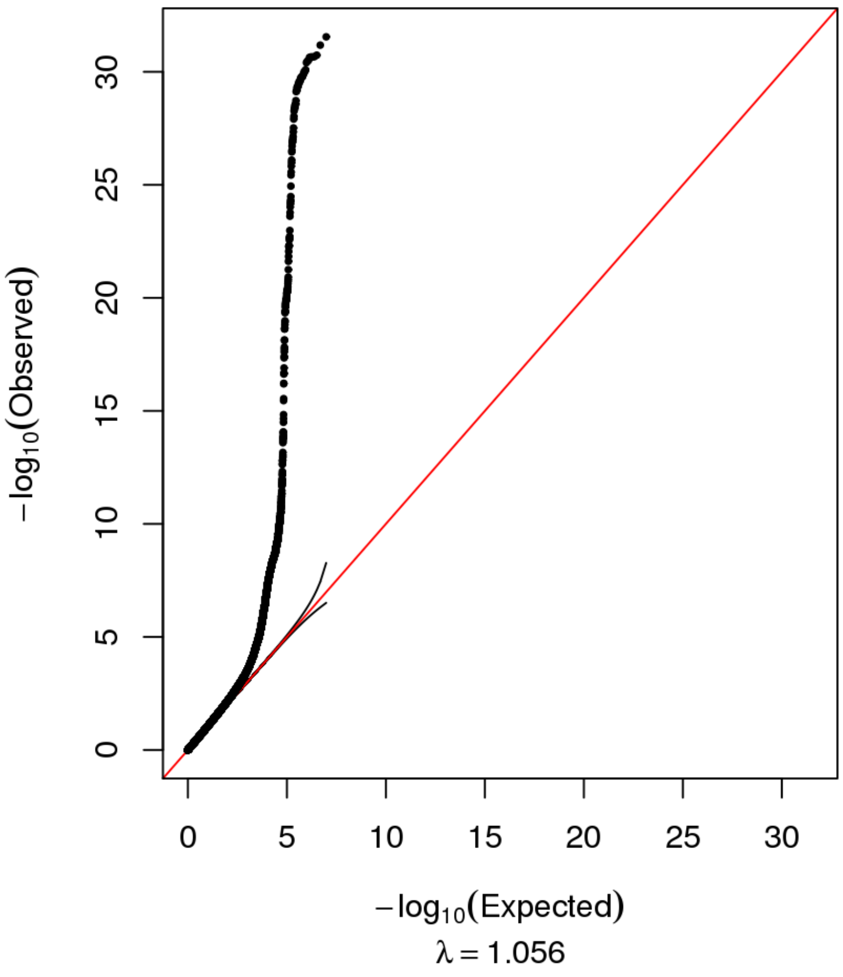

b.

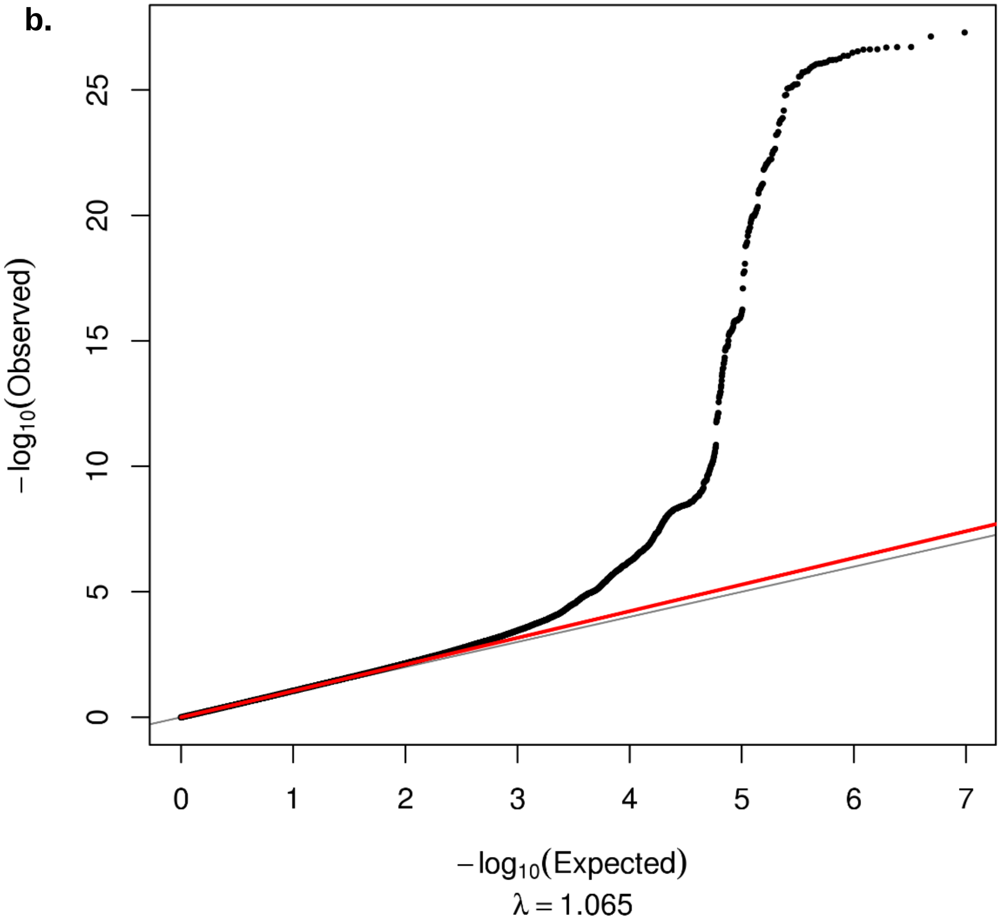

c.

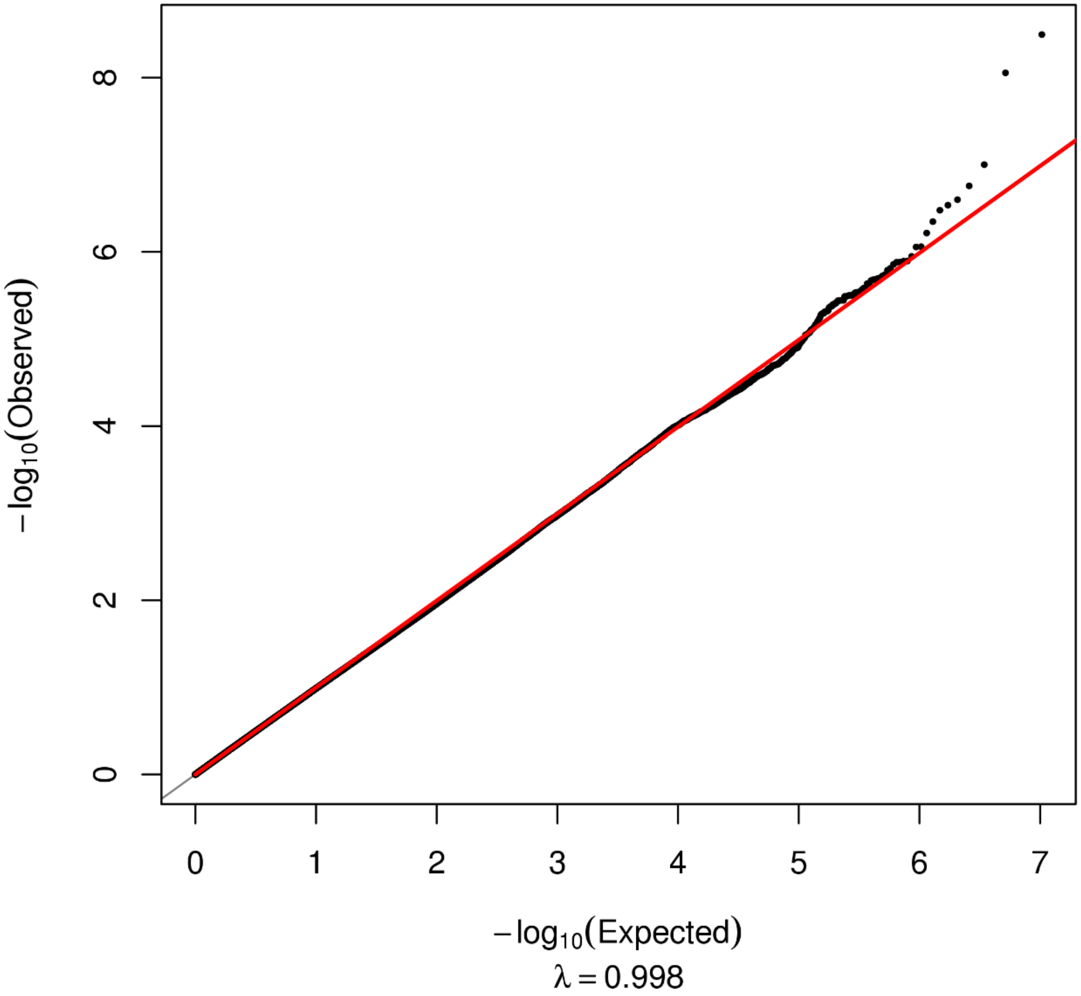

d.

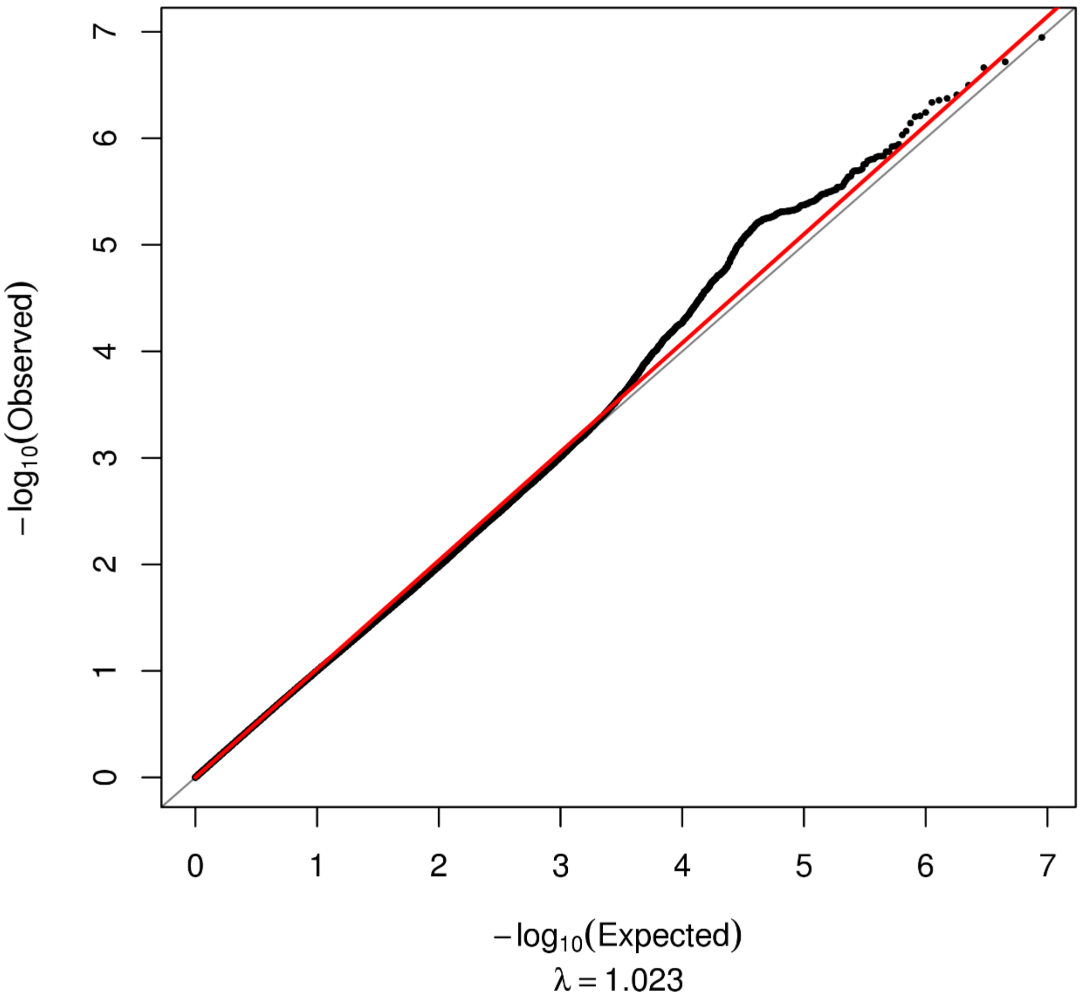

e.

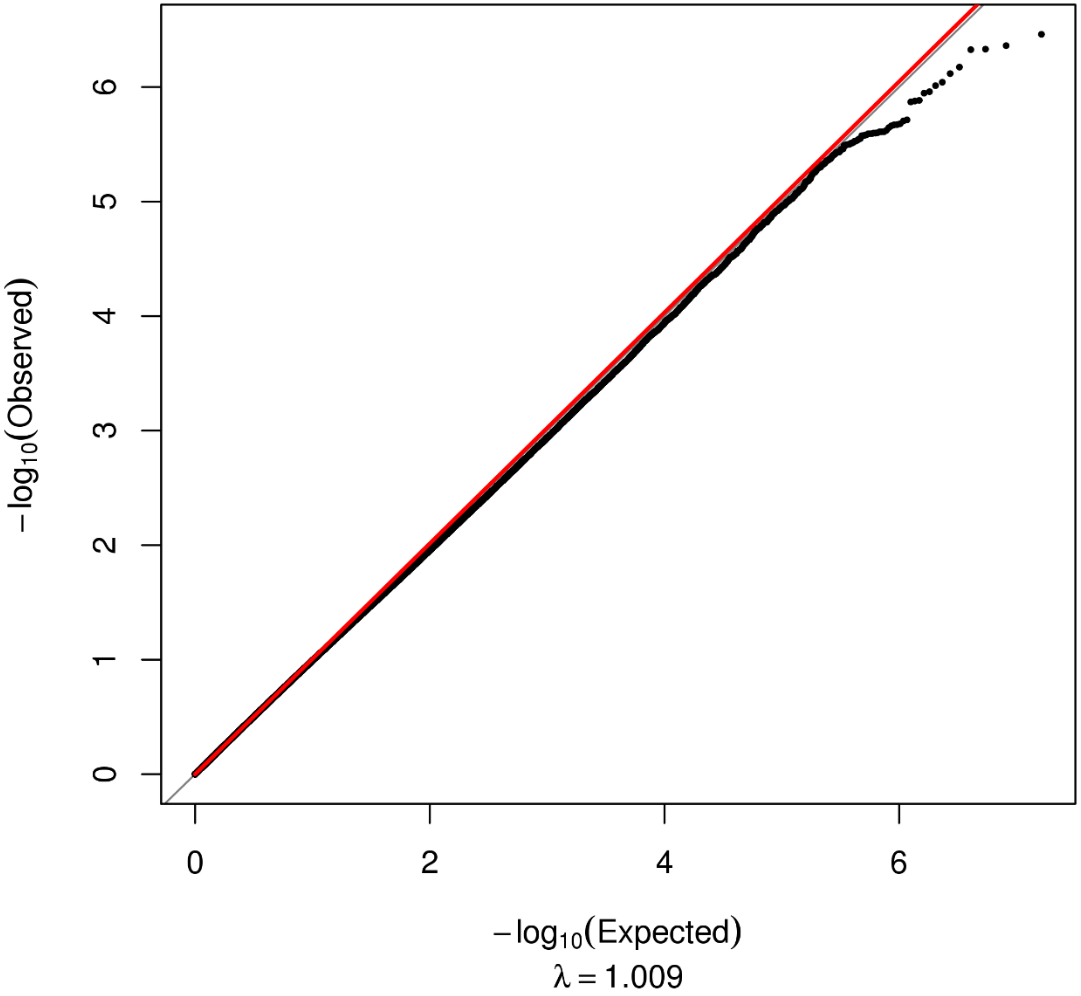

0
1
2
3
4
5
6
$-\log_{10}(\text{Observed})$
0
2
4
6
$-\log_{10}(\text{Expected})$
$\lambda = 1.009$

Supplementary Figure 2. Chicago plot of the GERA GWAS meta-analysis of POAG (upper panel) and UKB GWAS meta-analysis of glaucoma (lower panel)

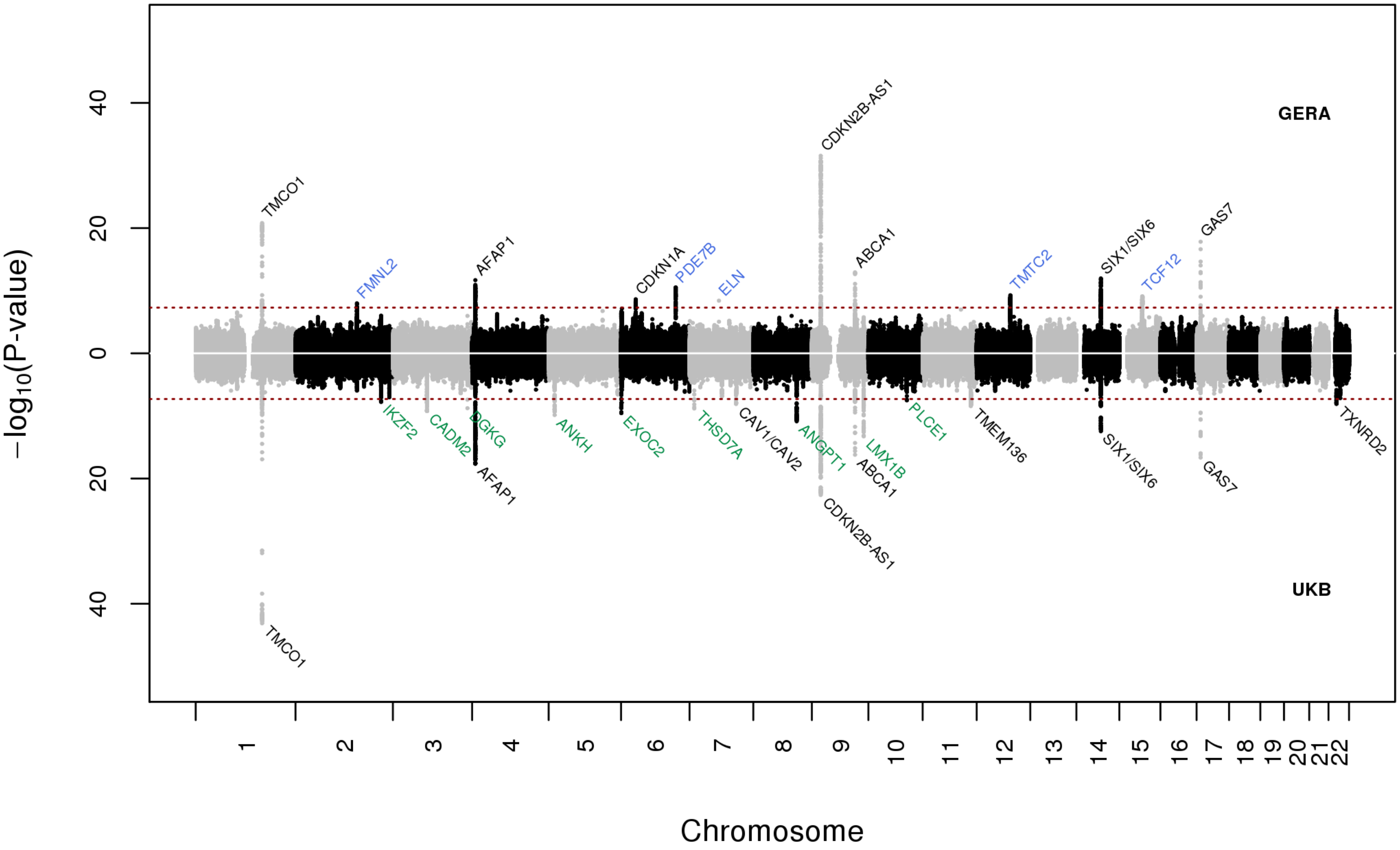

Association results (–log10 *P*-values) are plotted for each chromosome. The locus/gene names are indicated in black for previously reported loci, in blue for each novel locus identified in GERA and associated with POAG, and in green for each novel locus identified in UKB and associated with glaucoma.

Supplementary Figure 3. Regional plots for the POAG-associated loci in the discovery GERA multiethnic meta-analysis. Regional plots for the loci near: **a.** *FMNL2*, **b.** *PDE7B*, **c.** near *ELN*, **d.** *TMTC2*, and **e.** *TCF12* which reached genome-wide significance in the combined meta-analysis. "h19/1000 Genomes 2014 EUR" was used as the Genome Build/LD Population.

a.

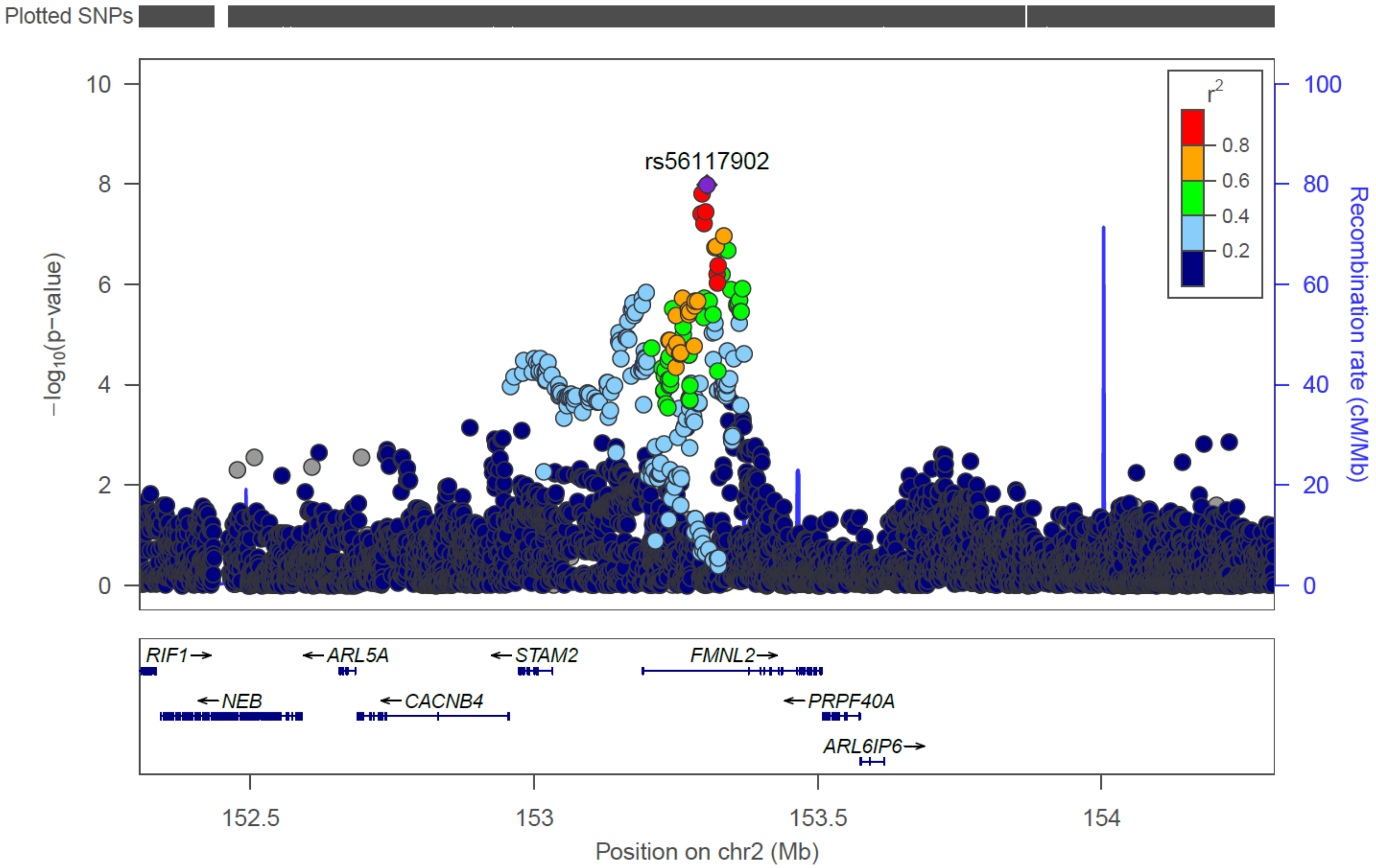

b.

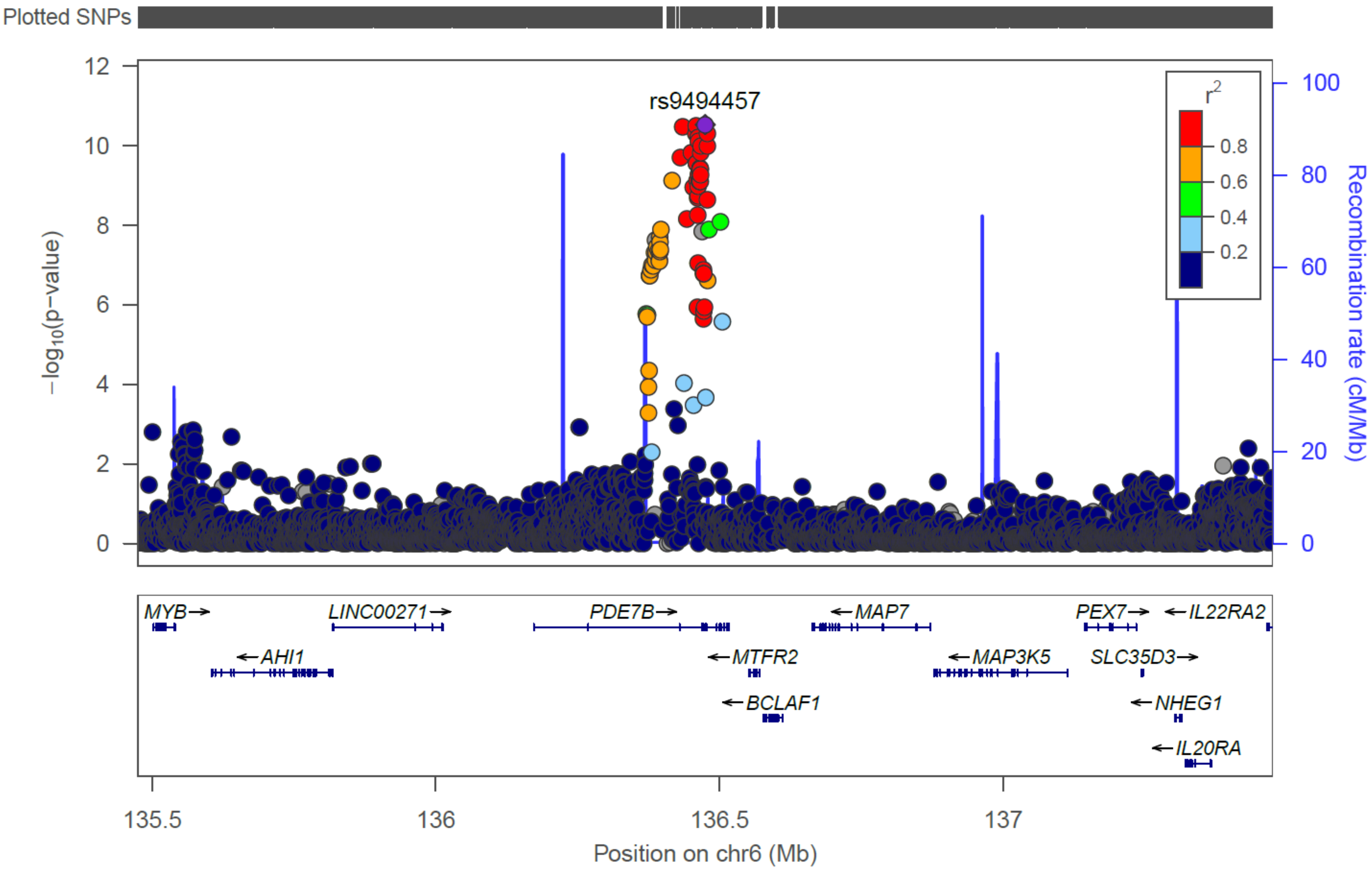
Plotted SNPs
rs9494457
−log10(p−value)
Recombination rate (cM/Mb)
r2
0.8
0.6
0.4
0.2
MYB
AHI1
LINC00271
PDE7B
MTFR2
BCLAF1
MAP7
MAP3K5
PEX7
SLC35D3
IL22RA2
NHEG1
IL20RA
135.5
136
136.5
137
Position on chr6 (Mb)

c.

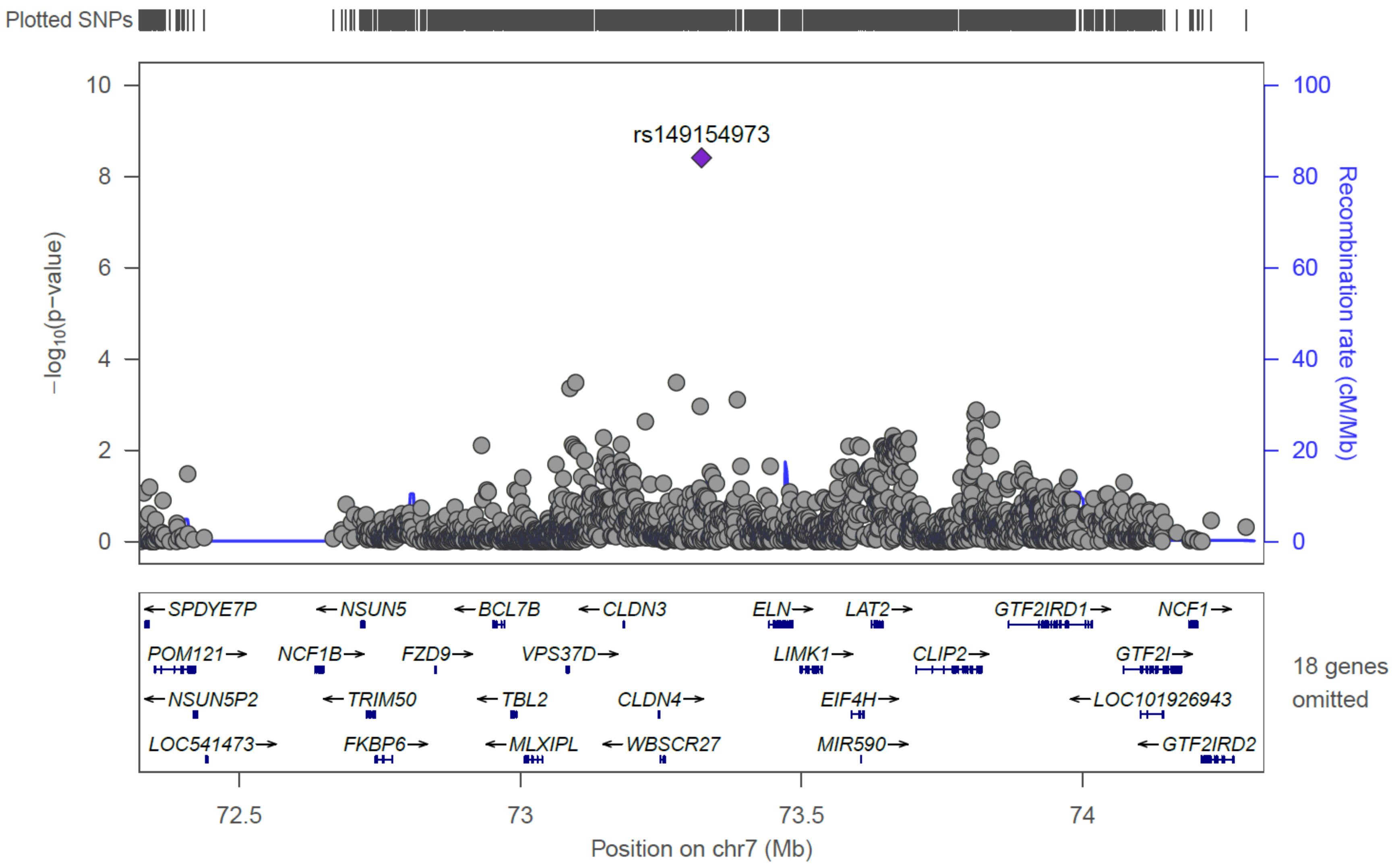

Plotted SNPs
10
8
6
4
2
0
−log10(p−value)
rs149154973
100
80
60
40
20
0
Recombination rate (cM/Mb)
←SPDYE7P
←NSUN5
←BCL7B
←CLDN3
ELN→
LAT2→
GTF2IRD1→
NCF1→
POM121→
NCF1B→
FZD9→
VPS37D→
LIMK1→
CLIP2→
GTF2I→
←NSUN5P2
←TRIM50
←TBL2
CLDN4→
EIF4H→
←LOC101926943
LOC541473→
FKBP6→
←MLXIPL
←WBSCR27
MIR590→
←GTF2IRD2
18 genes omitted
72.5
73
73.5
74
Position on chr7 (Mb)

d.

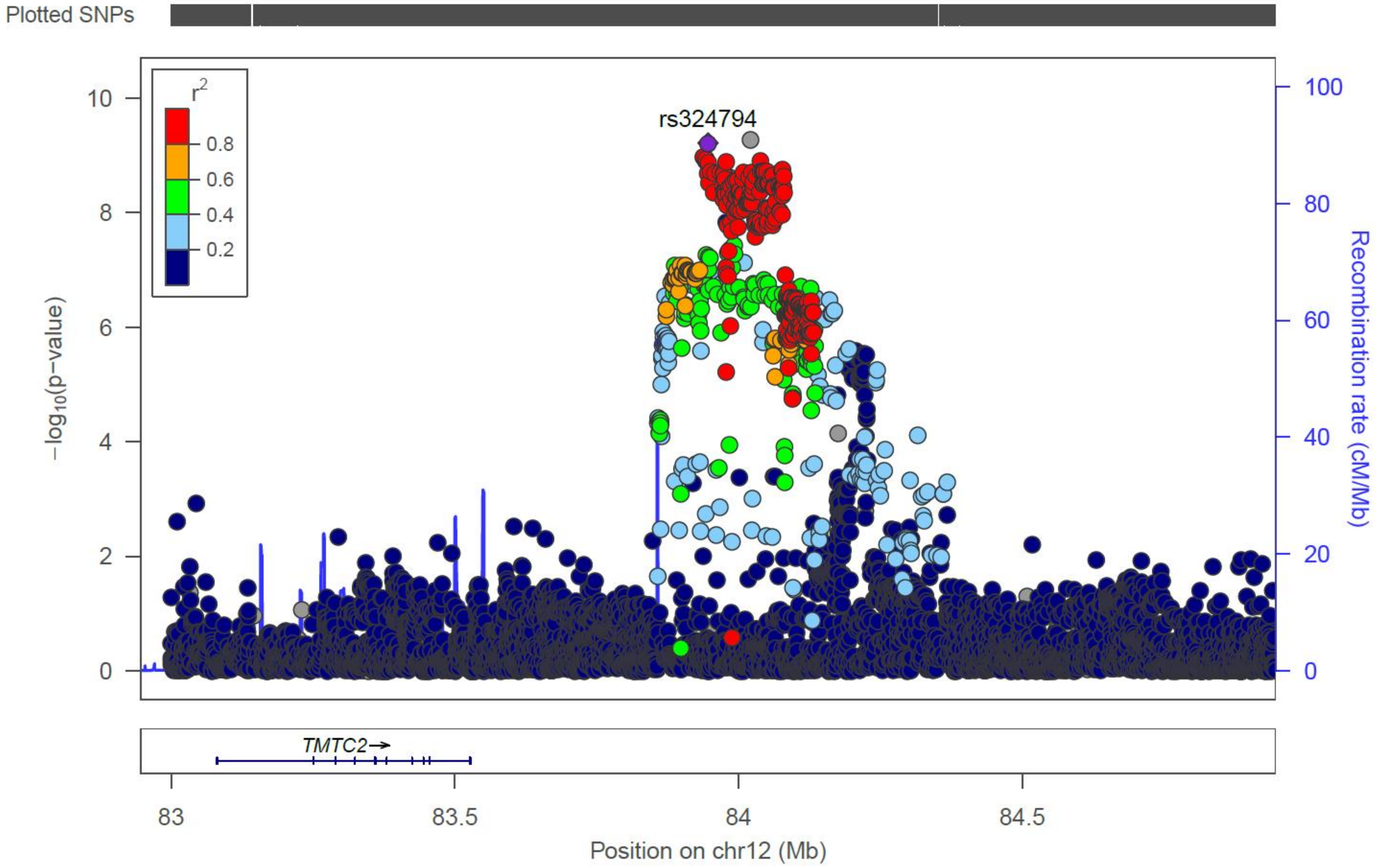
Plotted SNPs
r2
0.8
0.6
0.4
0.2
rs324794
−log10(p−value)
10
8
6
4
2
0
100
80
60
40
20
0
Recombination rate (cM/Mb)
TMTC2→
83
83.5
84
84.5
Position on chr12 (Mb)

e.

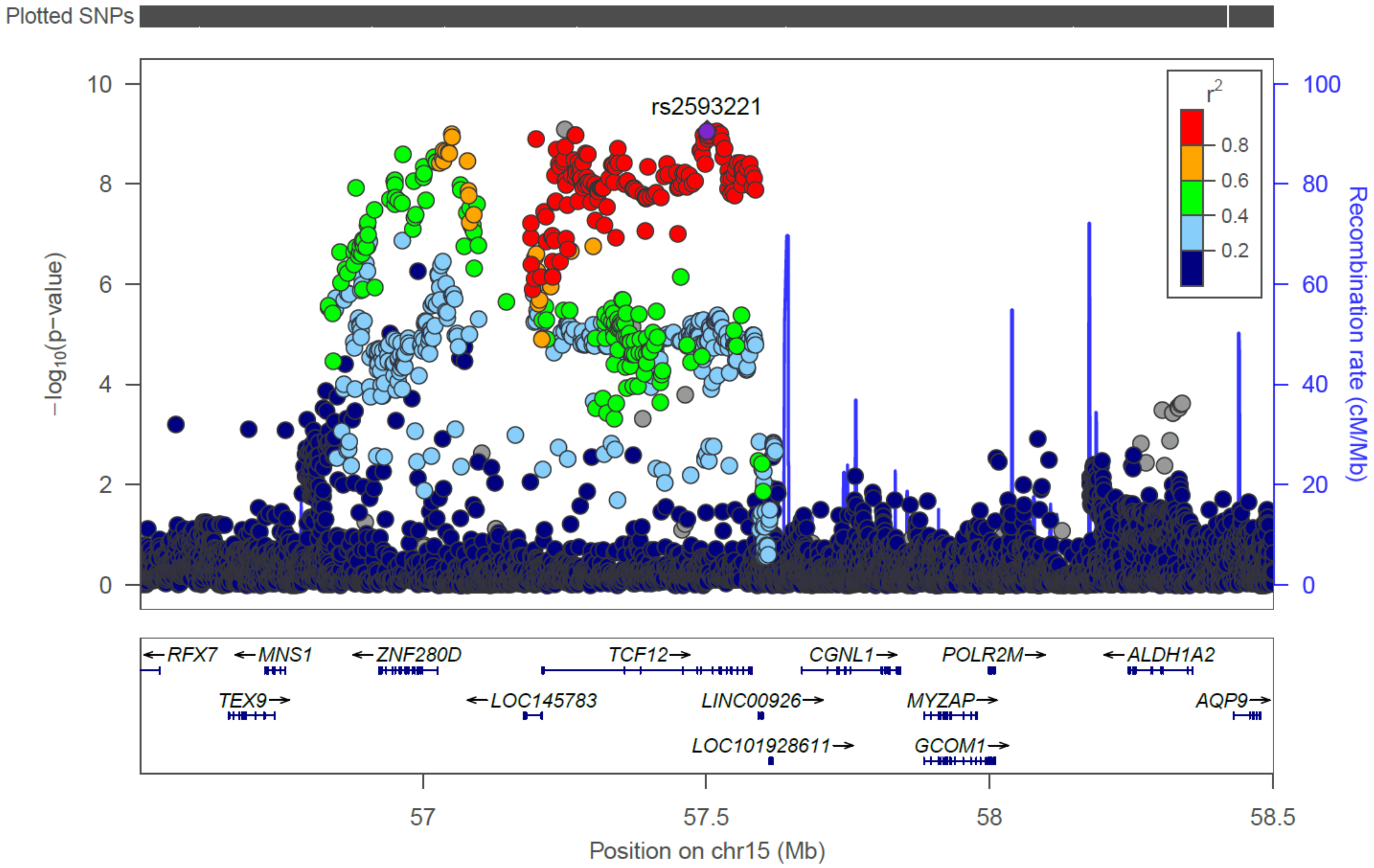

Plotted SNPs
rs2593221
r2
0.8
0.6
0.4
0.2
10
8
6
4
2
0
−log10(p−value)
100
80
60
40
20
0
Recombination rate (cM/Mb)
←RFX7
←MNS1
←ZNF280D
TCF12→
CGNL1→
POLR2M→
←ALDH1A2
TEX9→
←LOC145783
LINC00926→
MYZAP→
AQP9→
LOC101928611→
GCOM1→
57
57.5
58
58.5
Position on chr15 (Mb)

Supplementary Figure 4. Expression of glaucoma candidate genes in retinal ganglion cells (RGCs) in a mouse model of inherited glaucoma

a. Individual gene expression plots showing log_2 fold change of transcripts in DBA/2J2 (D2) RGCs of genes identified in the credible set analysis. D2 and D2-*Gpnmb*$^+$ mice used for the gene expression studies were from the The Jackson Laboratory. Experimental mice were all 9 month old females (See GEO entry: GSE90654). Bars represent individual transcripts, grey = not differentially expressed, red = differentially expressed at a FDR (Bonferroni adjusted), q <0.05 compared to D2-*Gpnmb*$^+$ controls (as in Williams *et al.* Science 2017[1]). There are no differentially expressed genes in Group 1 (representing samples that are transcriptomically identical to D2-*Gpnmb*$^+$ controls and have not yet progressed to glaucoma). Replicates used for each group are as follows: D2-*Gpnmb*$^+$ Control group = 9, D2 Group 1 = 6, Group 2 = 5, Group 3 = 10, Group 4 = 4.

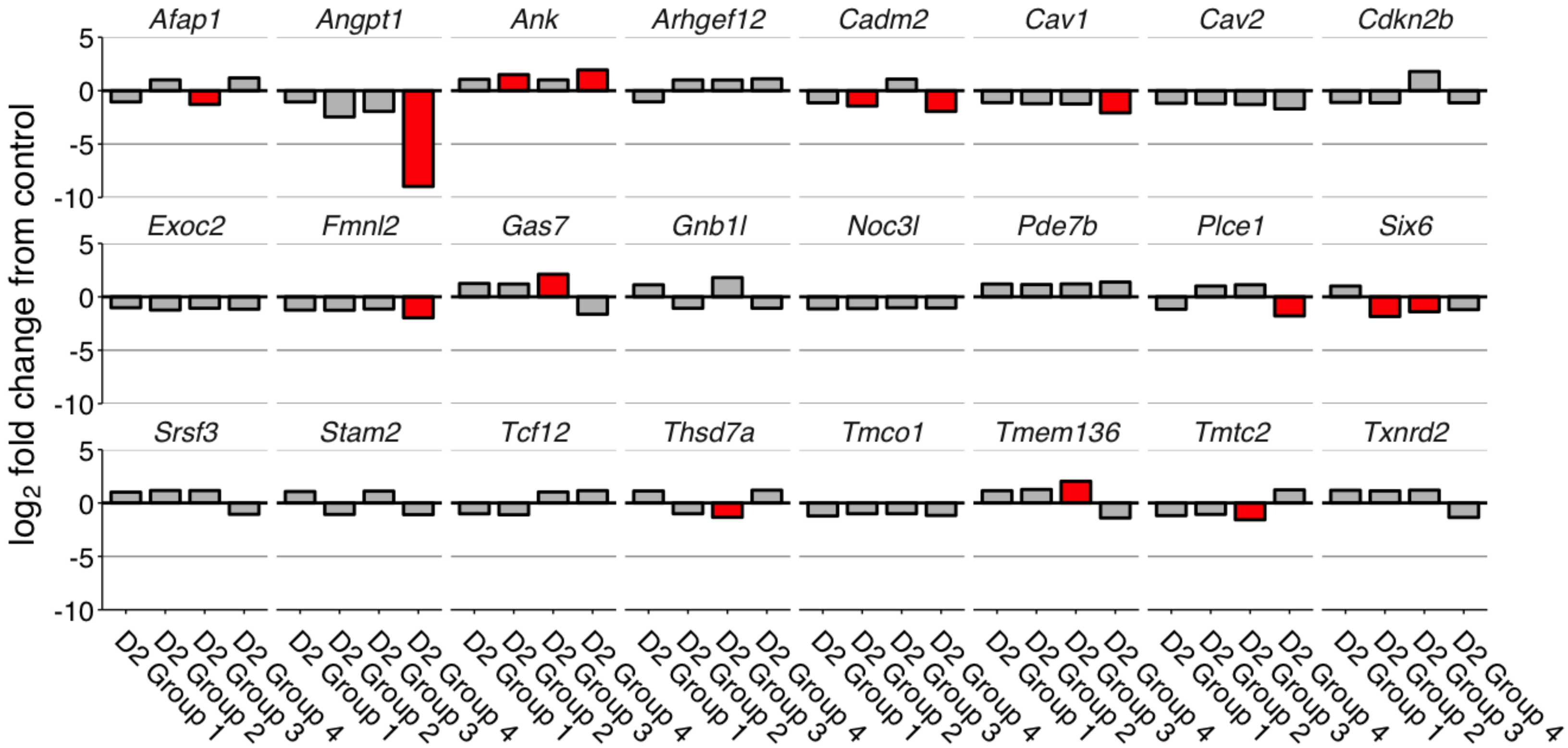

b. Normalized counts per million (CPM) of transcript levels of genes identified in the credible set analysis. Normalization was performed using the trimmed mean of M values (TMM) method.

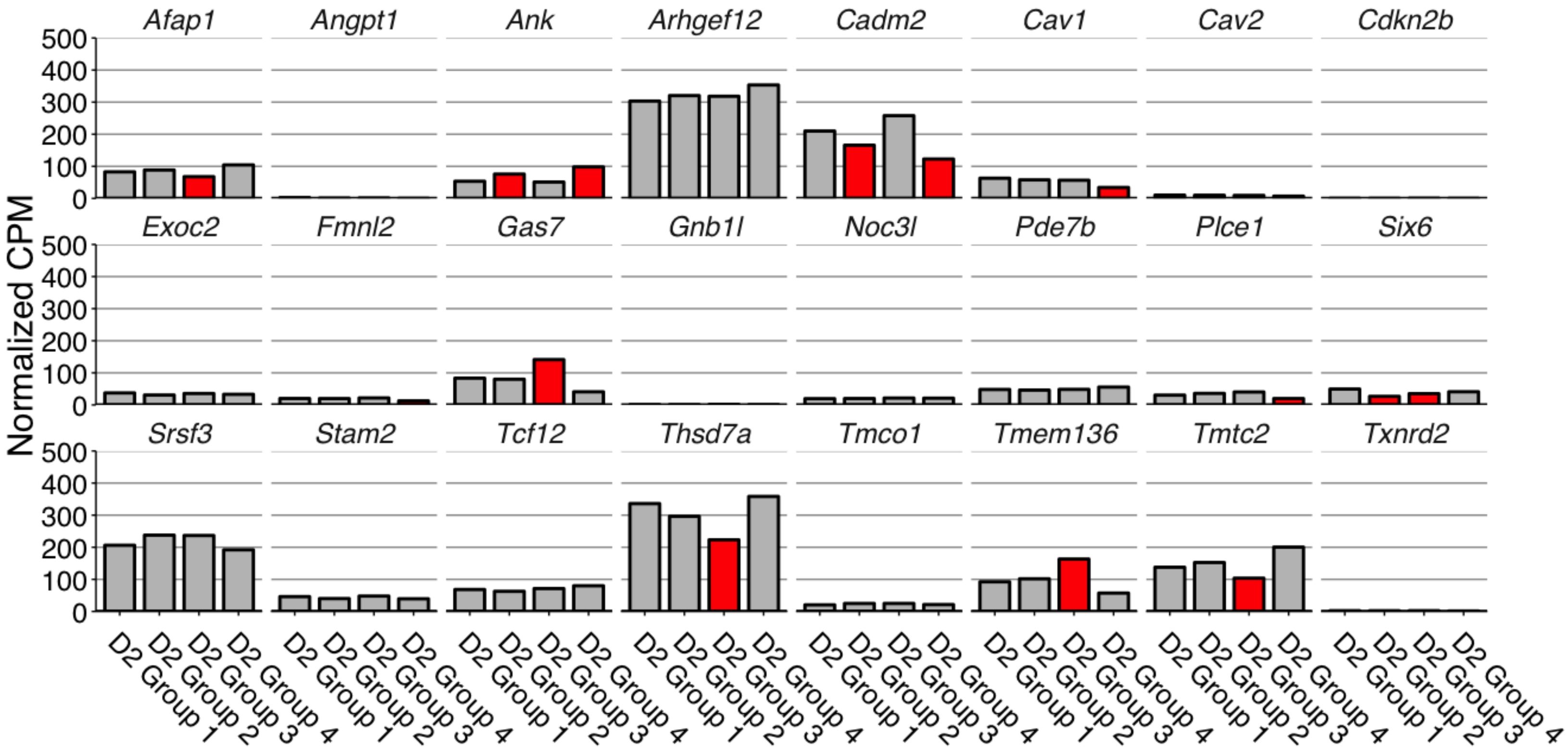

Supplementary Figure 5. *FMNL2* knockdown induces change in human trabecular meshwork cell morphology. *Fmnl2* was silenced in the HTM using siRNA2. The cells were then serum-starved for 24 hours to disrupt the normal actin assembly. Followed by FBS treatment for 10 minutes or 1 hour to induce formation of actin stress fibers and cell spreading. **a.** Fluorescent microscopic images of Phalloidin-FITC stained F-actin shows that the actin stress fibers are poorly organized in the serum starved control siRNA transfected cells (-FBS), and following treatment with FBS (+FBS for 10 min or 1hour) the stress fibers are prominently visible. Moreover, the control siRNA transfected HTM cells treated with FBS for 10 minutes or 1 hour are well spread. In contrast, the actin stress fibers of the HTM cells transfected with FMNL2-siRNA2 exhibited poorly formed actin stress fiber even after 1hour treatment with FBS. Furthermore, in general the cells appear poorly spread. **b.** Magnified images of Phalloidin-FITC stained F-actin presented in **a** are shown. White arrows indicate cells with deformed/rounded morphology, arrowheads indicate cells with modest spresding (intermediate) and asterisks indicate well-spread cells. Scale = 50 μm.

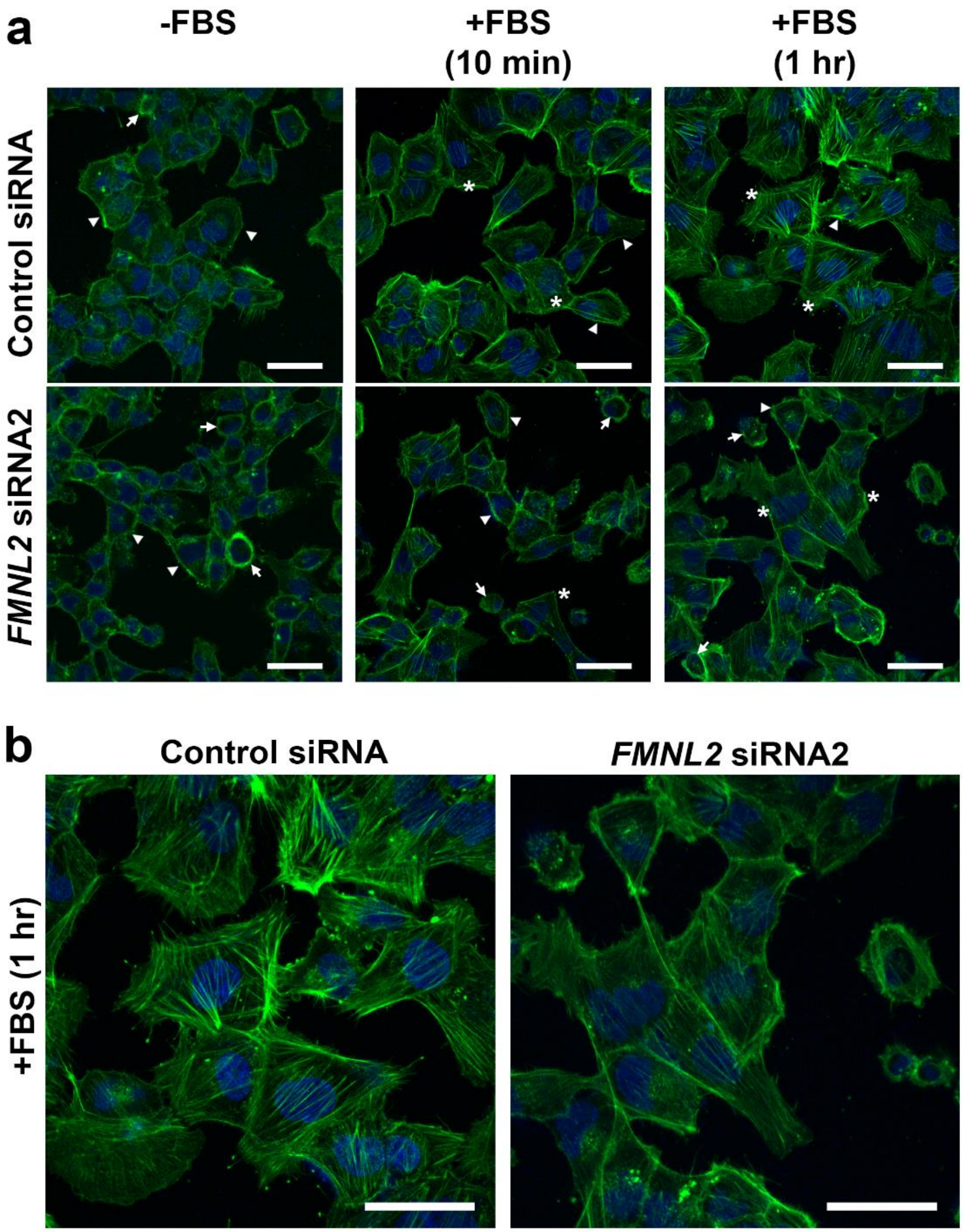
a
-FBS
+FBS (10 min)
+FBS (1 hr)
Control siRNA
FMNL2 siRNA2
b
Control siRNA
FMNL2 siRNA2
+FBS (1 hr)

Supplementary Table 1. Association of each covariate (age, sex, ancestry PCs) with POAG in the GERA cohort. For the interpretation of the PCs, see Banda *et al.* Genetics 2015[2]. Effect size estimates (β) and p-values are from the multivariate regression model.

**Non-Hispanic whites**			
	**β**	**SE**	***P*-value**
Age	0.07	0.002	$< 9.2 \times 10^{-308}$
Sex	-0.06	0.03	0.09
PC1	4.30	2.33	0.06
PC2	-0.42	2.50	0.87
PC3	0.97	2.78	0.73
PC4	3.38	2.91	0.24
PC5	-2.91	3.33	0.38
PC6	0.04	2.90	0.99
PC7	-2.62	3.13	0.40
PC8	-0.20	3.13	0.95
PC9	-0.09	3.06	0.98
PC10	1.16	3.07	0.71
ASHK	0.08	0.11	0.50
**Hispanic/Latinos**			
	**β**	**SE**	***P*-value**
Age	0.06	0.005	1.7×10^{-34}
Sex	-0.07	0.11	0.54
PC1	-34.78	6.43	6.3×10^{-8}
PC2	3.17	6.21	0.61
PC3	-4.91	5.76	0.39
PC4	-2.81	6.01	0.64
PC5	5.39	4.41	0.22
PC6	7.72	4.72	0.10
**East Asians**			
	**β**	**SE**	***P*-value**
Age	0.06	0.005	8.8×10^{-38}
Sex	-0.40	0.11	0.00015
PC1	8.53	5.86	0.15
PC2	25.13	4.67	7.3×10^{-8}
PC3	6.67	4.91	0.17
PC4	1.74	5.36	0.75
PC5	2.44	4.61	0.60

PC6	9.32	6.75	0.17
**African Americans**			
	**β**	**SE**	***P*-value**
Age	0.07	0.007	6.78×10^{-28}
Sex	-0.26	0.13	0.05
PC1	10.37	3.98	0.01
PC2	1.24	3.73	0.74
PC3	2.92	3.67	0.43
PC4	-0.15	3.67	0.97
PC5	1.38	3.74	0.71
PC6	0.82	3.75	0.83

Abbreviations: PC, principal component; ASHK, Ashkenazi ancestry proportion; β, beta; SE, standard error

Supplementary Table 2. Lead POAG SNPs (P < 5 x 10^{-8}) by race/ethnicity group in the GERA discovery cohort

			NHW			H/L			EAS			AFR			META	
**SNP**	**Locus**	**Alleles**	**Frq**	**OR (95%CI)**	***P***	**Frq**	**OR (95%CI)**	***P***	**Frq**	**OR (95%CI)**	***P***	**Frq**	**OR (95%CI)**	***P***	***P*het**	**I^2**
rs7524755	*TMCO1*	T/C	0.12	1.33 (1.24-1.43)	**1.4 x 10^{-16}**	0.13	1.51 (1.25-1.82)	2.4 x 10^{-5}	0.02	0.91 (0.47-1.77)	0.79	0.12	1.44 (1.11-1.86)	0.0053	0.39	0.9
rs56117902	*FMNL2*	A/C	0.46	0.88 (0.84-0.92)	2.6 x 10^{-7}	0.54	0.92 (0.79-1.06)	0.24	0.94	0.85 (0.62-1.17)	0.32	0.79	0.76 (0.61-0.95)	0.017	0.58	0.0
rs59521811	*AFAP1*	T/C	0.68	0.84 (0.80-0.89)	**2.7 x 10^{-11}**	0.59	0.84 (0.73-0.98)	0.026	0.66	0.94 (0.81-1.10)	0.44	0.56	0.98 (0.81-1.18)	0.84	0.28	21.5
rs6913530	*near CDKN1A*	G/A	0.78	0.87 (0.82-0.92)	7.0 x 10^{-7}	0.81	0.69 (0.58-0.83)	5.9 x 10^{-5}	0.62	0.92 (0.80-1.07)	0.29	0.88	0.90 (0.68-1.18)	0.43	0.08	55.7
rs9494457	*PDE7B*	T/A	0.61	1.17 (1.11-1.23)	**1.0 x 10^{-9}**	0.58	1.25 (1.07-1.45)	0.0050	0.66	1.03 (0.88-1.20)	0.71	0.63	1.16 (0.95-1.42)	0.16	0.37	4.3
rs149154973	*near ELN*	TG/T	0.96	0.70 (0.62-0.79)	**1.6 x 10^{-8}**	0.97	0.85 (0.54-1.31)	0.45	0.98	1.08 (0.56-2.11)	0.82	0.99	0.47 (0.22-0.99)	0.047	0.41	0.0
rs10811645	*CDKN2B-AS1*	G/A	0.51	0.77 (0.73-0.81)	**2.4 x 10^{-27}**	0.34	0.78 (0.66-0.92)	0.0024	0.15	0.68 (0.55-0.85)	0.0008	0.31	0.83 (0.68-1.02)	0.070	0.64	0.0
rs2472493	*near ABCA1*	G/A	0.42	1.19 (1.13-1.25)	**7.5 x 10^{-13}**	0.37	1.16 (1.00-1.35)	0.046	0.56	0.99 (0.86-1.15)	0.90	0.35	1.25 (1.04-1.51)	0.018	0.11	49.7
rs324794	*near TMTC2*	G/T	0.45	0.86 (0.82-0.91)	**1.9 x 10^{-9}**	0.41	0.94 (0.81-1.09)	0.38	0.16	0.88 (0.71-1.07)	0.20	0.32	0.90 (0.73-1.10)	0.31	0.77	0.0
rs35155027	*SIX1/SIX6*	G/C	0.41	1.18 (1.12-1.24)	**4.2 x 10^{-11}**	0.41	1.10 (0.95-1.28)	0.20	0.77	1.35 (1.12-1.64)	0.0021	0.84	1.03 (0.81-1.30)	0.82	0.26	25.4
rs2593221	*TCF12*	A/G	0.76	0.86 (0.82-0.91)	**4.5 x 10^{-8}**	0.76	0.85 (0.72-1.00)	0.053	0.83	0.80 (0.67-0.96)	0.019	0.71	0.96 (0.79-1.18)	0.71	0.63	0.0
rs9913911	*GAS7*	A/G	0.62	1.24 (1.18-1.30)	**6.9 x 10^{-17}**	0.67	1.17 (1.00-1.37)	0.055	0.52	1.09 (0.94-1.26)	0.26	0.79	1.37 (1.07-1.75)	0.012	0.27	23.9

Abbreviations: NHW, non-Hispanic whites; H/L, Hispanic/Latinos; EAS, East Asians; AA, African-Americans; Frq, frequency of effect allele; OR, odds ratio; P-values in bold achieved genome-wide level of significance (P < 5 x 10^{-8}); loci highlighted in grey are novel; *P*het, heterogeneity *P*-values from Cochrane's Q statistic; I^2, heterogeneity index (0–100%)

Supplementary Table 3. Characteristics of the glaucoma cases and controls from UK Biobank

		Controls	Cases
**Total**	N, (proportion that are cases)	169,561	7,329 (4.1%)
**Sex**	Women,<br>N, (proportion that are cases)	92,327	3,460 (3.6%)
	Men<br>N, (proportion that are cases)	77,234	3,869 (4.8%)
**Race/Ethnicity**	European<br>N, (proportion that are cases)	155,514	6,687 (4.1%)
	South Asian<br>N, (proportion that are cases)	4,916	204 (4.0%)
	African British<br>N, (proportion that are cases)	4,831	272 (5.3%)
	Mixed Ancestries<br>N, (proportion that are cases)	3,427	144 (4.0%)
	East Asian<br>N, (proportion that are cases)	873	22 (2.5%)
Age (years) Mean ± SD		58.0 ± 8.2	62.0 ± 6.4

Abbreviations: N, number; SD, standard deviation; Age, age when glaucoma was first diagnosed within cases and age at recruitment within controls

Supplementary Table 4. Conditional analysis results from the GERA multiethnic meta-analysis

**SNP**	**Chr**	**Pos**	**Locus**	**Alleles**	**OR (95%CI)**	***P***
rs74899281	1	165827908	*UCK2*	AG/A	0.52 (0.39-0.71)	2.5×10^{-5}
rs2069418	9	22009698	*CDKN2B-AS1*	G/C	0.90 (0.86-0.95)	1.4×10^{-5}
rs76958481	14	61125237	*near SIX1*	C/T	0.51 (0.36-0.71)	9.1×10^{-5}

Abbreviations: SNP, single nucleotide polymorphism; Chr, chromosome; Pos, position; OR, odds ratio; *P*, *P*-values

Supplementary Table 5. Comparison of the effects of lead SNPs from the GERA discovery GWAS for POAG, HTG, NTG, and IOP

				**POAG**		**IOP**		**NTG**		**HTG**	
**SNP**	**Chr**	**Locus**	**Alleles**	**OR (95%CI)**	***P***	**β (95%CI)**	***P***	**OR (95%CI)**	***P***	**OR (95%CI)**	***P***
High IOP loci											
rs7524755	1	*TMCO1*	T/C	1.35 (1.27-1.44)	**1.6 x 10^{-21}**	0.22 (0.19, 0.29)	**1.7 x 10^{-19}**	1.09 (0.91-1.30)	0.34	1.39 (1.31-1.49)	**1.4 x 10^{-23}**
rs56117902	2	*FMNL2*	A/C	0.88 (0.84-0.92)	**1.0 x 10^{-8}**	-0.07 (-0.11, -0.04)	2.1 x 10^{-6}	0.99 (0.88-1.11)	0.84	0.86 (0.82-0.91)	**1.2 x 10^{-9}**
rs59521811	4	*AFAP1*	T/C	0.86 (0.82-0.90)	**1.2 x 10^{-11}**	-0.13 (-0.16, -0.10)	**3.6 x 10^{-15}**	0.86 (0.77-0.96)	5.9 x 10^{-3}	0.86 (0.82-0.90)	**3.2 x 10^{-10}**
rs9494457	6	*PDE7B*	T/A	1.16 (1.11-1.22)	**3.0 x 10^{-11}**	0.07 (0.04, 0.10)	1.7 x 10^{-6}	1.07 (0.96-1.19)	0.22	1.18 (1.13-1.24)	**1.2 x 10^{-11}**
rs2472493	9	*near ABCA1*	G/A	1.17 (1.12-1.22)	**1.2 x 10^{-13}**	0.17 (0.14, 0.20)	**1.1 x 10^{-28}**	1.13 (1.02-1.25)	0.02	1.18 (1.13-1.23)	**1.6 x 10^{-12}**
rs9913911	17	*GAS7*	A/G	1.22 (1.17-1.28)	**1.5 x 10^{-18}**	0.21 (0.18, 0.24)	**1.4 x 10^{-42}**	1.12 (1.00-1.25)	0.04	1.24 (1.18-1.30)	**1.3 x 10^{-18}**
Normal IOP Loci											
rs6913530	6	*near CDKN1A*	G/A	0.86 (0.82-0.90)	**2.3 x 10^{-9}**	-0.04 (-0.07, 0.00)	0.032	0.75 (0.67-0.84)	8.8 x 10^{-7}	0.88 (0.84-0.93)	5.9 x 10^{-6}
rs149154973	7	*near ELN*	TG/T	0.70 (0.62-0.79)	**3.9 x 10^{-9}**	-0.01 (-0.10, 0.08)	0.87	0.66 (0.49-0.90)	9.3 x 10^{-3}	0.71 (0.63-0.80)	7.7 x 10^{-8}
rs10811645	9	*CDKN2B-AS1*	G/A	0.77 (0.74-0.80)	**2.8 x 10^{-32}**	0.01 (-0.02, 0.04)	0.65	0.61 (0.54-0.69)	**6.2 x 10^{-17}**	0.80 (0.76-0.83)	**1.0 x 10^{-21}**
rs324794	12	*near TMTC2*	G/T	0.87 (0.83-0.91)	**6.1 x 10^{-10}**	-0.02 (-0.05, 0.01)	0.22	0.83 (0.74-0.93)	1.1 x 10^{-3}	0.88 (0.84-0.92)	5.6 x 10^{-8}
rs35155027	14	*SIX1/SIX6*	G/C	1.17 (1.12-1.23)	**1.1 x 10^{-12}**	-0.01 (-0.04, 0.02)	0.43	1.37 (1.22-1.54)	4.7 x 10^{-8}	1.14 (1.09-1.20)	**2.8 x 10^{-8}**
rs2593221	15	*TCF12*	A/G	0.86 (0.82-0.90)	**8.5 x 10^{-10}**	-0.05 (-0.09, -0.02)	0.0028	0.84 (0.75-0.95)	4.0 x 10^{-3}	0.86 (0.82-0.91)	**2.1 x 10^{-8}**

Abbreviations: OR, odds ratio; *P*-values in bold achieved genome-wide level of significance ($P < 5 \times 10^{-8}$); POAG, primary open angle glaucoma; HTG, high tension glaucoma; NTG, normal tension glaucoma; IOP, intraocular pressure; loci highlighted in grey are novel. Here, we report the lead SNP for each independent locus identified in the GERA discovery GWAS of POAG. Note: "High IOP loci" were defined as loci significantly associated with IOP (higher or lower) at a Bonferroni level of significance (P<0.0014 for 12 SNPs in 3 analyses). In contrast, "Normal IOP loci" were defined as loci not associated with IOP (meaning with a P≥0.0014).

Supplementary Table 6. Replication of SNP associations reported in previous NTG GWAS studies in the GERA discovery cohort

					Meta-analysis				NHW		H/L		EAS		AA	
**Chr**	**SNP**	**Nearest Gene**	**Ref**	**Alleles**	**OR (95%CI)**	***P***	***P*het**	***I*²**	**OR (95%CI)**	***P***	**OR (95%CI)**	***P***	**OR (95%CI)**	***P***	**OR (95%CI)**	***P***
8	rs284491	-	[3,4]	C/T	1.06 (0.95-1.19)	0.29	0.43	0.0	1.12 (0.97-1.29)	0.11	1.05 (0.73-1.51)	0.79	0.99 (0.79-1.25)	0.94	0.75 (0.46-1.24)	0.26
9	rs1333037	*CDKN2B-AS1*	[3,4]	C/T	0.59 (0.52-0.67)	**1.3×10^{-16}**	0.99	0.0	0.59 (0.51-0.68)	**1.3×10^{-13}**	0.58 (0.36-0.92)	0.02	0.58 (0.39-0.85)	0.01	0.68 (0.27-1.73)	0.42
12	rs2041895	*TMEM263*	[3]	C/G	1.04 (0.94-1.16)	0.43	0.50	0.0	0.98 (0.86-1.12)	0.81	1.13 (0.79-1.59)	0.51	1.20 (0.95-1.51)	0.12	1.10 (0.63-1.93)	0.72

Abbreviations: NHW, non-Hispanic whites; H/L, Hispanic/Latinos; EAS, East Asians; AA, African Americans; *P*-values in bold achieved genome-wide level of significance ($P < 5 \times 10^{-8}$); *P*het, heterogeneity *P*-values from Cochrane's Q statistic; I^2, heterogeneity index (0–100%)

Supplementary Table 7. Replication of SNP associations reported in previous POAG GWAS studies in the GERA cohort

				**Meta-analysis**				**NHW**		**H/L**		**EAS**		**AA**	
**SNP**	**Nearest Gene**	**Ref**	**Alleles**	**OR (95%CI)**	***P***	***P*het**	***I*²**	**OR (95%CI)**	***P***	**OR (95%CI)**	***P***	**OR (95%CI)**	***P***	**OR (95%CI)**	***P***
rs1192415	*CDC7–TGFBR3*	5	G/A	1.12 (1.06-1.18)	2.4×10^{-5}	0.80	0.0	1.13 (1.06-1.20)	0.00011	1.17 (0.99-1.38)	0.06	1.05 (0.86-1.29)	0.61	1.04 (0.81-1.33)	0.75
rs4656461	*TMCO1*	6	G/A	1.33 (1.25-1.41)	**1.2×10^{-19}**	0.57	0.0	1.33 (1.25-1.43)	**1.3×10^{-16}**	1.42 (1.17-1.71)	0.00029	0.95 (0.49-1.84)	0.88	1.22 (0.97-1.53)	0.09
rs7588567	*NCKAP5*	7	T/C	NA	NA	NA	NA	0.98 (0.92-1.04)	0.45	0.91 (0.76-1.09)	0.32	1.03 (0.89-1.20)	0.65	0.86 (0.68-1.10)	0.23
rs4619890	*AFAP1*	8	G/A	1.08 (1.04-1.13)	0.00025	0.23	29.6	1.08 (1.03-1.13)	0.0015	1.22 (1.05-1.42)	0.0092	0.97 (0.84-1.14)	0.75	1.10 (0.88-1.39)	0.40
rs2745572	*FOXC1*	3	A/G	1.12 (1.07-1.18)	5.2×10^{-7}	0.01	73.1	1.16 (1.10-1.22)	**1.8×10^{-8}**	0.98 (0.84-1.14)	0.80	0.94 (0.81-1.09)	0.43	1.29 (1.01-1.65)	0.05
rs11969985	*GMDS*	8	G/A	1.13 (1.06-1.20)	0.00017	0.21	33.1	1.17 (1.09-1.26)	2.4×10^{-5}	1.10 (0.89-1.36)	0.38	0.99 (0.83-1.19)	0.94	0.98 (0.79-1.22)	0.85
rs67530707	*near CDKN1A*	9	TCA/T	0.86 (0.82-0.91)	**3.7×10^{-9}**	0.10	52.4	0.87 (0.82-0.92)	8.3×10^{-7}	0.70 (0.58-0.84)	9.7×10^{-5}	0.92 (0.79-1.07)	0.27	0.92 (0.70-1.22)	0.57
rs4236601	*CAV1/CAV2*	10	G/A	0.91 (0.87-0.95)	1.0×10^{-4}	0.71	0.0	0.91 (0.86-0.96)	0.00042	0.96 (0.81-1.14)	0.66	0.77 (0.49-1.21)	0.25	0.85 (0.70-1.04)	0.11
rs4977756	*CDKN2B-AS1*	6	G/A	0.81 (0.77-0.84)	**9.7×10^{-21}**	0.01	75.0	0.79 (0.75-0.83)	**4.3×10^{-20}**	0.74 (0.62-0.89)	0.00093	0.87 (0.73-1.03)	0.10	1.11 (0.91-1.36)	0.29
rs2472493	*ABCA1*	8	G/A	1.17 (1.12-1.22)	**1.2×10^{-13}**	0.11	49.7	1.19 (1.13-1.25)	**7.5×10^{-13}**	1.16 (1.00-1.35)	0.046	0.99 (0.86-1.15)	0.90	1.25 (1.04-1.51)	0.018
rs542340	*DNAJC24*	11	A/G	NA	NA	NA	NA	NA	NA	NA	NA	NA	NA	0.68 (0.32-1.47)	0.33
rs58073046	*ARHGEF12*	12	A/G	0.87 (0.82-0.93)	4.3×10^{-5}	0.81	0.0	0.86 (0.80-0.92)	4.6×10^{-5}	0.97 (0.73-1.30)	0.86	0.91 (0.75-1.10)	0.33	0.94 (0.56-1.56)	0.80
rs7137828	*ATXN2*	3	C/T	0.93 (0.89-0.98)	0.0034	0.18	39.4	0.93 (0.89-0.98)	0.0033	0.97 (0.83-1.14)	0.73	0.40 (0.17-0.92)	0.030	1.05 (0.78-1.41)	0.74
rs199748604	*TRIM9-TMX1*	11	T/TG	1.01 (0.92-1.12)	0.80	0.06	64.4	1.02 (0.92-1.13)	0.70	1.25 (0.79-1.98)	0.34	0.80 (0.50-1.26)	0.33	0.48 (0.25-0.94)	0.031
rs10483727	*SIX1/SIX6*	4	T/C	1.17 (1.12-1.22)	**1.6×10^{-12}**	0.22	31.6	1.18 (1.12-1.23)	**6.3×10^{-11}**	1.11 (0.96-1.28)	0.17	1.35 (1.12-1.64)	0.0020	1.00 (0.79-1.27)	0.97
rs192917960	*RBFOX1*	11	C/T	NA	NA	NA	NA	NA	NA	NA	NA	NA	NA	1.19 (0.52-2.70)	0.68
rs3785176	*PMM2*	13	A/C	0.98 (0.93-1.03)	0.35	0.77	0.0	0.99 (0.93-1.04)	0.61	1.00 (0.82-1.20)	0.96	0.91 (0.77-1.06)	0.22	0.93 (0.70-1.23)	0.61
rs9897123	*GAS7*	3	C/T	1.18 (1.13-1.23)	**1.1×10^{-13}**	0.35	9.2	1.20 (1.14-1.26)	**1.1×10^{-13}**	1.08 (0.93-1.26)	0.31	1.08 (0.93-1.26)	0.33	1.13 (0.93-1.37)	0.23
rs35934224	*TXNRD2*	3	C/T	1.15 (1.08-1.22)	2.8×10^{-5}	0.60	0.0	1.17 (1.09-1.26)	2.9×10^{-5}	1.04 (0.84-1.28)	0.72	1.27 (0.87-1.84)	0.22	1.06 (0.85-1.31)	0.60

Abbreviations: Chr, chromosome; SNP, single nucleotide polymorphism; NHW, non-Hispanic whites; H/L, Hispanic/Latinos; EAS, East Asians; AA, African Americans; *P*-values in bold achieved genome-wide level of significance ($P < 5 \times 10^{-8}$); *P*het, heterogeneity *P*-values from Cochrane's Q statistic; I^2, heterogeneity index (0–100%); *NCKAP5* rs7588567 had a moderate imputation quality score r^2 in non-Hispanic whites (r^2=0.64), as well as in Hispanic/Latinos (r^2=0.63) and in African-Americans (r^2=0.61). For this reason, rs7588567 was excluded from the meta-analysis across the 4 race/ethnicity groups.

Supplementary Table 8. Lead SNPs ($P < 5 \times 10^{-8}$) in the multiethnic meta-analysis (GERA+UKB)

					Meta-analysis (GERA+UKB)				GERA		UKB	
**Chr**	**Pos**	**SNP**	**Nearest Gene**	**Alleles**	**OR (95%CI)**	***P***	***P*het**	I^2	**OR (95%CI)**	***P***	**OR (95%CI)**	***P***
1	88227120	rs41461152	*LMO4/PKN2-AS1*	T/G	1.09 (1.06-1.12)	4.1×10^{-9}	0.27	17.3	1.11 (1.06-1.16)	5.7×10^{-6}	1.07 (1.04-1.11)	9.6×10^{-5}
1	103385373	rs993471	*COL11A1*	G/A	1.08 (1.05-1.11)	2.0×10^{-8}	0.98	0	1.08 (1.03-1.13)	0.00055	1.08 (1.04-1.12)	9.9×10^{-6}
1	165739598	rs2814471	*TMCO1*	C/T	1.37 (1.32-1.42)	2.0×10^{-62}	0.42	0	1.34 (1.26-1.43)	2.0×10^{-20}	1.39 (1.32-1.45)	7.5×10^{-44}
2	55933014	rs2627761	*near PNPT1*	C/T	1.11 (1.07-1.15)	6.1×10^{-10}	0.32	0.5	1.13 (1.07-1.19)	3.0×10^{-6}	1.09 (1.05-1.14)	2.9×10^{-5}
2	66567896	rs2860235	*near MEIS1*	T/C	0.93 (0.90-0.95)	3.9×10^{-8}	0.55	0	0.94 (0.90-0.98)	0.0033	0.92 (0.89-0.95)	2.9×10^{-6}
2	111680155	rs3789134	*ACOXL*	T/C	1.09 (1.06-1.12)	1.9×10^{-9}	0.87	0	1.09 (1.04-1.14)	0.00012	1.09 (1.05-1.12)	4.0×10^{-6}
2	153357541	rs6434068	*FMNL2*	G/C	0.91 (0.89-0.94)	1.9×10^{-11}	0.44	0	0.90 (0.86-0.94)	2.4×10^{-6}	0.92 (0.89-0.95)	1.2×10^{-6}
2	213758234	rs56335522	*IKZF2*	G/C	1.19 (1.13-1.24)	1.7×10^{-13}	0.75	0	1.20 (1.11-1.29)	2.0×10^{-6}	1.18 (1.11-1.25)	1.7×10^{-8}
2	234271522	rs34339006	*DGKD*	C/T	0.93 (0.90-0.95)	1.7×10^{-8}	0.14	54	0.95 (0.91-0.99)	0.019	0.91 (0.88-0.94)	9.4×10^{-8}
3	25574186	rs1153606	*RARB*	A/G	1.10 (1.06-1.14)	3.4×10^{-8}	0.97	0	1.10 (1.04-1.16)	0.00046	1.10 (1.05-1.15)	2.0×10^{-5}
3	85138818	rs9883252	*CADM2*	T/C	1.11 (1.08-1.14)	6.1×10^{-14}	0.69	0	1.10 (1.05-1.15)	2.0×10^{-5}	1.11 (1.08-1.15)	6.2×10^{-10}
3	150063454	rs73162480	*TSC22D2*	G/T	1.15 (1.09-1.20)	1.8×10^{-8}	0.81	0	1.15 (1.07-1.25)	0.00043	1.14 (1.08-1.21)	1.1×10^{-5}
3	186131600	rs9853115	*DGKG/TBCCD1*	T/A	1.10 (1.08-1.13)	1.9×10^{-13}	0.7	0	1.10 (1.05-1.14)	2.2×10^{-5}	1.11 (1.07-1.15)	1.7×10^{-9}
3	188066437	rs6771736	*LPP*	C/G	0.92 (0.89-0.95)	4.9×10^{-8}	0.78	0	0.92 (0.88-0.97)	0.0012	0.92 (0.88-0.95)	1.1×10^{-5}
4	7916540	rs6857814	*AFAP1*	A/G	0.86 (0.84-0.89)	5.8×10^{-27}	0.96	0	0.86 (0.83-0.90)	4.9×10^{-11}	0.86 (0.83-0.89)	1.8×10^{-17}
5	14837332	rs76325372	*ANKH*	A/C	1.12 (1.08-1.15)	6.7×10^{-13}	0.18	45.2	1.09 (1.04-1.14)	0.00045	1.14 (1.09-1.18)	1.4×10^{-10}
5	172588027	rs255303	*BNIP1*	G/T	1.08 (1.05-1.11)	3.5×10^{-8}	0.22	32.2	1.06 (1.01-1.10)	0.017	1.09 (1.06-1.13)	3.0×10^{-7}
6	642017	rs72835984	*EXOC2*	C/T	0.86 (0.83-0.89)	1.7×10^{-15}	0.96	0	0.86 (0.81-0.92)	1.1×10^{-6}	0.86 (0.82-0.90)	2.9×10^{-10}
6	1989604	rs9392348	*GMDS*	G/A	1.11 (1.08-1.15)	1.7×10^{-10}	0.13	55.6	1.15 (1.09-1.21)	2.9×10^{-7}	1.09 (1.05-1.14)	4.4×10^{-5}
6	51406848	rs17752199	*near PKHD1*	A/G	1.14 (1.09-1.19)	4.7×10^{-9}	0.07	69.3	1.20 (1.12-1.29)	3.9×10^{-7}	1.10 (1.04-1.17)	0.00059
6	136458593	rs3757155	*PDE7B*	C/T	1.12 (1.09-1.15)	9.5×10^{-15}	0.021	81.3	1.16 (1.11-1.22)	3.2×10^{-11}	1.09 (1.05-1.13)	4.0×10^{-6}
6	158976277	rs4709212	*TMEM181*	C/A	1.09 (1.06-1.12)	7.8×10^{-9}	0.5	0	1.08 (1.03-1.13)	0.0017	1.10 (1.06-1.14)	1.0×10^{-6}
6	170448016	rs3012455	*near FAM120B*	A/G	1.14 (1.09-1.20)	9.3×10^{-9}	0.82	0	1.15 (1.06-1.24)	0.00042	1.14 (1.08-1.20)	5.7×10^{-6}
7	11677452	rs2526101	*THSD7A*	A/G	0.92 (0.89-0.94)	1.4×10^{-10}	0.14	53.1	0.94 (0.90-0.98)	0.0056	0.90 (0.87-0.93)	2.5×10^{-9}
7	80848822	rs327636	*SEMA3C/CACNA2D1*	A/G	1.08 (1.06-1.12)	7.0×10^{-9}	0.43	0	1.07 (1.02-1.12)	0.0030	1.09 (1.06-1.13)	4.7×10^{-7}
7	116154831	rs6969706	*CAV2/CAV1*	G/T	0.90 (0.88-0.93)	3.8×10^{-12}	0.68	0	0.91 (0.87-0.95)	9.6×10^{-5}	0.90 (0.86-0.93)	8.4×10^{-9}
7	117632975	rs6947612	*CTTNBP2/LSM8*	A/G	0.92 (0.90-0.95)	1.2×10^{-8}	0.64	0	0.93 (0.89-0.97)	0.0017	0.92 (0.89-0.95)	1.7×10^{-6}
7	151505698	rs62478245	*PRKAG2*	C/T	0.82 (0.76-0.88)	3.3×10^{-8}	0.98	0	0.82 (0.71-0.93)	0.0025	0.82 (0.75-0.89)	3.7×10^{-6}

8	108275781	rs2514882	*ANGPT1*	C/T	0.87 (0.84-0.91)	7.3×10^{-12}	0.025	80	0.92 (0.87-0.98)	0.0072	0.84 (0.80-0.88)	2.2×10^{-11}
8	124552133	rs62521287	*FBXO32*	C/T	0.86 (0.81-0.90)	8.3×10^{-9}	0.69	0	0.84 (0.77-0.92)	0.00026	0.86 (0.81-0.92)	7.5×10^{-6}
9	22051670	rs944801	*CDKN2B-AS1*	G/C	0.81 (0.79-0.83)	2.1×10^{-49}	0.0017	89.9	0.76 (0.73-0.80)	2.0×10^{-30}	0.84 (0.81-0.87)	7.4×10^{-23}
9	107695848	rs2472493	*near ABCA1*	G/A	1.16 (1.13-1.19)	6.1×10^{-29}	0.57	0	1.17 (1.12-1.22)	1.2×10^{-13}	1.15 (1.12-1.19)	6.3×10^{-17}
9	129382560	rs1536907	*LMX1B*	A/G	0.87 (0.84-0.90)	1.1×10^{-17}	0.39	0	0.89 (0.84-0.94)	2.5×10^{-5}	0.86 (0.83-0.90)	6.6×10^{-14}
10	60374898	rs2393455	*BICC1*	C/A	1.08 (1.05-1.11)	7.1×10^{-9}	0.66	0	1.09 (1.04-1.14)	8.3×10^{-5}	1.08 (1.04-1.11)	2.0×10^{-5}
10	96015793	rs3891783	*PLCE1*	C/G	0.93 (0.90-0.95)	1.6×10^{-8}	0.08	67.2	0.95 (0.92-1.00)	0.031	0.91 (0.88-0.94)	3.6×10^{-8}
11	47345100	rs10838692	*MADD*	T/C	0.92 (0.89-0.94)	8.8×10^{-10}	0.44	0	0.91 (0.87-0.95)	1.1×10^{-5}	0.93 (0.89-0.96)	1.4×10^{-5}
11	65225319	rs12808303	*near NEAT1*	C/G	1.12 (1.08-1.16)	3.5×10^{-10}	0.32	0	1.14 (1.08-1.21)	4.8×10^{-6}	1.10 (1.06-1.15)	1.0×10^{-5}
11	120207405	rs7126413	*near ARHGEF12*	A/G	0.91 (0.89-0.94)	1.1×10^{-11}	0.45	0	0.92 (0.88-0.96)	0.00037	0.90 (0.87-0.94)	5.2×10^{-9}
12	84038478	rs12309467	*TMTC2/SLC6A15*	C/G	1.08 (1.06-1.11)	1.9×10^{-9}	0.0024	89.2	1.14 (1.10-1.19)	1.2×10^{-9}	1.05 (1.02-1.09)	0.0037
13	22669058	rs17075855	*LINC00540*	G/A	0.91 (0.88-0.94)	3.8×10^{-8}	0.74	0	0.90 (0.86-0.95)	0.00016	0.91 (0.87-0.95)	6.1×10^{-5}
14	61091401	rs34935520	*SIX1/SIX6*	G/A	1.15 (1.12-1.18)	7.8×10^{-24}	0.27	17.5	1.17 (1.12-1.22)	1.8×10^{-12}	1.14 (1.10-1.17)	3.6×10^{-13}
15	57086199	rs1647381	*ZNF280D/TCF12*	C/G	0.90 (0.87-0.93)	8.2×10^{-9}	0.017	82.4	0.85 (0.80-0.90)	5.7×10^{-8}	0.93 (0.89-0.97)	0.0021
15	61952501	rs2245899	*near VPS13C*	G/A	1.08 (1.05-1.11)	5.1×10^{-9}	0.25	24.5	1.10 (1.06-1.15)	5.8×10^{-6}	1.07 (1.03-1.11)	0.00011
17	10031183	rs9913911	*GAS7*	A/G	1.19 (1.15-1.22)	9.1×10^{-34}	0.12	59.6	1.22 (1.17-1.28)	1.5×10^{-18}	1.17 (1.13-1.21)	2.2×10^{-17}
20	6473123	rs6140010	*near CASC20*	A/G	1.09 (1.06-1.12)	7.6×10^{-10}	0.42	0	1.11 (1.06-1.16)	8.4×10^{-6}	1.08 (1.04-1.12)	1.5×10^{-5}
22	19856710	rs58714937	*near TXNRD2*	C/T	1.15 (1.11-1.20)	1.1×10^{-13}	0.95	0	1.15 (1.09-1.23)	2.6×10^{-6}	1.15 (1.10-1.21)	8.3×10^{-9}
22	29105610	rs5752774	*CHEK2*	C/T	0.91 (0.88-0.93)	1.8×10^{-11}	0.86	0	0.90 (0.86-0.95)	2.1×10^{-5}	0.91 (0.88-0.94)	1.9×10^{-7}

Abbreviations: Chr, chromosome; Pos, position; SNP, single nucleotide polymorphism; OR, odds ratio; P-values in bold achieved genome-wide level of significance (P < 5 x 10-8); loci highlighted in grey are additional novel glaucoma loci identified in the multi-ethnic meta-analysis combining the GERA and UKB cohorts; *P*het, heterogeneity *P*-values from Cochrane's Q statistic; I^2, heterogeneity index (0–100%)

Supplementary Table 9. Associations of genetic ancestries before and after adjusting for previously reported and newly identified SNPs

	Before adjustment			After adjustment for 67 SNPs		
**Non-Hispanic whites**						
	**β**	**SE**	***P*-value**	**β**	**SE**	***P*-value**
PC1: Northwest vs. Southeast	4.30	2.33	0.06	3.00	2.31	0.19
PC2: Northeast vs. Southwest	-0.42	2.50	0.87	-0.24	2.49	0.92
GRS	-	-	-	0.63	0.02	1.16×10^{-200}
**Hispanic/Latinos**						
	**β**	**SE**	***P*-value**	**β**	**SE**	***P*-value**
PC1: European vs. Native American	-34.78	6.43	6.3×10^{-8}	-33.74	6.46	1.74×10^{-7}
PC2: Other vs. African	3.17	6.21	0.61	6.57	6.19	0.29
GRS	-	-	-	0.62	0.067	1.25×10^{-20}
**East Asians**						
	**β**	**SE**	***P*-value**	**β**	**SE**	***P*-value**
PC1: East Asian vs. European	8.53	5.86	0.15	4.51	5.94	0.45
PC2: North vs. South	25.13	4.67	7.3×10^{-8}	28.72	4.67	7.85×10^{-10}
GRS	-	-	-	0.66	0.09	7.39×10^{-13}
**African Americans**						
	**β**	**SE**	***P*-value**	**β**	**SE**	***P*-value**
PC1: African vs. European	10.37	3.98	0.01	3.71	4.13	0.37
PC2: East Asian vs. Other	1.24	3.73	0.74	1.22	3.81	0.75
GRS	-	-	-	0.70	0.098	1.16×10^{-12}

Abbreviations: PC, principal component; GRS, genetic risk score; β, effect size estimates; SE, standard error; the 67 SNPs include previously reported and newly identified in GERA, UKB, and meta-analysis GERA+UKB

Supplementary Table 10. Expression of the genes in each identified locus that contained associated 95% credible set variants in adult human eye tissues

		The Ocular Tissue Database[14]											EyeSAGE[15,16]			
Chr	Nearest Gene	Probe ID	Choroid RPE	Ciliary Body	Cornea	Iris	Lens	Optic Nerve	Optic Nerve Head	Retina	Sclera	TM	TM	MAC	RPE MAC	RPE Peri
1	*TMCO1*	2442134	205.4	138.5	224.6	138.8	149.5	143.4	205.3	145.4	119.1	188.4	+	NA	NA	NA
2	*STAM2*	2581430	128.1	113.3	139.6	105.3	236.4	163.1	229.7	151.8	123.2	161.9	+	NA	NA	NA
2	*FMNL2*	2510713	154.1	64.7	41	121	181.7	338.4	394.5	33.1	186.5	77.2	+	-	+	+
3	*CADM2*	2631556	35.6	45.6	49.2	36.1	68.6	61.0	46.8	97.2	39.4	44.4	NA	NA	NA	NA
4	*AFAP1*	2717518	46.3	34.2	60.5	44.5	50.7	59.2	60.2	46.4	43.8	59.6	NA	NA	NA	NA
5	*ANKH*	2849469	78.9	70.1	89.6	53.9	150.7	76.8	114.7	83.8	164.1	112.6	+	+	+	+
6	*EXOC2*	2938196	35.9	25.5	29.8	29.9	24.4	28.0	35.3	35.2	36.7	32.3	+	NA	NA	NA
6	*SRSF3*	2905118	558.1	462.2	271.3	975.4	257.9	206.2	294.8	224.6	178.9	356.1	NA	NA	NA	NA
6	*PDE7B*	2926969	57.0	73.0	35.6	12.4	23.5	58.6	52.1	55.1	110.4	136.1	+	NA	NA	NA
7	*THSD7A*	NA	NA	NA	NA	NA	NA	NA	NA	NA	NA	NA	+	NA	NA	NA
7	*CAV2*	3020273	24.8	31.3	30.3	57.4	21.0	19.5	39.9	20.4	23.7	39.8	+	+	+	-
7	*CAV1*	3020302	37.1	49.6	68.6	95.4	283.6	37.6	81.0	35.3	48.4	98.4	+	+	+	+
8	*ANGPT1*	3148463	193.8	20.8	51.9	7.0	11.6	168.2	148.4	15.0	114.7	78.3	+	-	+	+
9	*CDKN2B-AS1*	3164914	35.9	32.7	37.0	29.7	22.5	33.3	33.2	26.0	31.7	33.4	+	NA	NA	NA
9	*LMX1B*	3189545	29.7	25.7	40.2	23.5	24.9	27.8	26.9	27.4	32.4	30.6	NA	-	+	-
10	*PLCE1*	3258477	132.5	104.7	26.8	26.4	172.4	165.9	167.7	60.6	140.1	143.2	+	+	+	+
10	*NOC3L*	3301011	48.6	40.7	47.6	37.6	83.2	39.0	49.2	36.4	36.2	50.7	+	NA	NA	NA
11	*TMEM136*	3352485	39.3	58.0	6.5	42.0	47.5	28.1	26.8	85.1	25.0	29.1	NA	NA	NA	NA
11	*ARHGEF12*	3352503	145.5	158.0	155.2	97.2	334.0	154.5	173.5	180.9	105.1	245.7	+	+	+	-
14	*C14orf39*	3567243	8.8	16.5	4.9	19.1	6.1	22.7	32.9	15.5	5.0	6.7	NA	+	-	-
14	*SIX6*	3538624	21.3	13.7	27.3	25.1	22.7	22.3	25.2	26.1	19.0	25.8	+	+	+	+
14	*LOC105378189*	NA	NA	NA	NA	NA	NA	NA	NA	NA	NA	NA	NA	NA	NA	NA
15	*ZNF280D*	3625823	84.5	69.4	63.3	103.0	77.4	92.9	92.7	83.5	73.8	116.1	NA	NA	NA	NA
15	*LOC1457*	NA	NA	NA	NA	NA	NA	NA	NA	NA	NA	NA	NA	NA	NA	NA
15	*TCF12*	3595096	96.1	63.0	133.7	92.0	91.7	96.1	101.2	73.1	96.5	126.9	+	+	-	+
17	*GAS7*	3744965	100.6	63.2	19.7	84.8	188.1	219.1	230.5	102.9	77.2	128.0	NA	+	+	+
22	*TXNRD2*	3952880	39.5	35.2	37.0	30.2	40.0	31.4	33.7	30.3	35.0	35.9	+	+	+	+
22	*GNB1L*	3952825	25.7	28.0	25.5	22.6	25.0	28.1	22.8	20.5	25.6	23.9	+	NA	NA	NA

Abbreviations: RPE, retinal pigment epithelium; TM, trabecular meshwork; MAC, retina macular; RPE Peri, RPE peripheral; na, not applicable. Genes highlighted in grey are in novel POAG loci identified in this study. In the Ocular Tissue Database[14] (OTDB), the gene expression is indicated as Affymetrix Probe Logarithmic Intensity Error (PLIER) number. The PLIER numbers were calculated by GC-background correction, PLIER normalization, log transformation and *z*-score calculation. The OTDB is also available at https://genome.uiowa.edu/otdb/. In the EyeSAGE[15,16] datasets from NEIBank, the gene expression is determined by tag counts in the Serial Analysis of Gene Expression (SAGE). All counts were summarized for each gene per tissue, and we added '+' to indicate the expression, or '-' to label the no expression while the counts are 0. The EyeSAGE is publicly available at the http://neibank.nei.nih.gov/EyeSAGE/index.shtml.

Supplementary Table 11. Expression pattern of glaucoma candidate genes in optic nerve head tissue

Gene ID	Log_2 (FC) Stage 1	QV Stage 1	Log_2 (FC) Stage 2	QV Stage 2	Log_2 (FC) Stage 3	QV Stage 3	Log_2 (FC) Stage 4	QV Stage 4	Log_2 (FC) Stage 5	QV Stage 5
Afap1	0.13	0.55	0.08	0.26	0.11	0.20	0.22	0.17	0.33	0.0073
Angpt1	-0.34	0.48	-0.01	0.56	-0.32	0.10	-0.46	0.12	0.30	0.11
Ank	0.13	0.55	0.15	0.20	0.00	0.54	0.27	0.14	0.34	0.033
Arhgef12	-0.03	0.75	-0.13	0.08	-0.18	0.06	-0.10	0.26	0.01	0.48
Cadm2	-0.29	0.16	-0.75	0.0022	-0.45	0.0203	-0.89	0.0078	-0.60	0.0074
Cav1	0.03	0.77	0.15	0.19	0.10	0.34	0.42	0.023	0.33	0.034
Cav2	0.14	0.55	0.16	0.12	0.25	0.07	0.27	0.11	0.43	0.0085
Cdkn2b	0.36	0.38	0.79	0.0017	1.26	0.0002	1.11	0.0018	1.72	0.0001
C14orf39	-0.52	0.25	-0.47	0.06	-0.40	0.09	-0.65	0.06	-0.50	0.08
Exoc2	0.03	0.73	-0.09	0.36	0.13	0.24	-0.07	0.47	-0.02	0.48
Fmnl2	0.07	0.65	0.14	0.05	0.11	0.27	0.17	0.19	0.21	0.038
Gas7	-0.08	0.75	-0.13	0.37	0.61	0.0088	0.44	0.05	0.14	0.31
Gnb1l	-0.06	0.70	0.20	0.12	0.05	0.46	-0.02	0.54	-0.01	0.48
Lmx1b	-0.14	0.54	0.02	0.52	-0.11	0.23	0.00	0.56	-0.06	0.38
Noc3l	0.12	0.61	-0.06	0.41	-0.09	0.37	0.17	0.23	0.21	0.11
Pde7b	0.08	0.71	0.41	0.0083	0.38	0.0475	0.30	0.06	0.12	0.28
Plce1	0.14	0.50	0.36	0.011	0.43	0.014	0.24	0.17	0.48	0.0060
Six6	-0.19	0.61	-0.17	0.28	0.32	0.06	-0.11	0.38	-0.09	0.32
Srsf3	-0.16	0.35	-0.37	0.0076	-0.32	0.0044	-0.22	0.09	-0.24	0.06
Stam2	0.14	0.55	0.20	0.15	0.35	0.043	0.37	0.07	0.39	0.06
Tcf12	0.13	0.49	0.19	0.08	0.27	0.023	0.32	0.06	0.48	0.0018
Tmco1	0.14	0.36	0.17	0.07	0.16	0.09	0.20	0.09	0.34	0.0045
Tmem136	-0.24	0.45	-0.45	0.0049	-0.17	0.24	-0.32	0.10	-0.14	0.24
Tmtc2	-0.22	0.62	-0.53	0.10	-0.88	0.0032	-0.70	0.025	-0.52	0.048
Txnrd2	-0.31	0.16	-0.21	0.19	-0.24	0.051	-0.33	0.0065	-0.67	0.0044

Data is from a previously published microarray gene expression dataset in optic nerve head punches[17,18]. Log_2 fold change (FC) is comparing DBA/2J mice relative to DBA/2J-*Gpnmb*$^+$ controls at 5 molecularly distinct stages of glaucoma defined using hierarchical clustering. Stages 1, 2 and 3 are early states of glaucoma that precede morphologically detectable glaucoma. Stages 4 and 5 have eyes with moderate and severe optic nerve damage respectively. Q-value (QV) shows the significance of the FC of GWAS genes at each stage as analyzed using MAANOVA.

Significant QV are highlighted in red. All the mice utilized for expression analysis were females. Replicates used for each group are as follows: D2-*Gpnmb*$^{+}$ Control = 10, D2 stage 1 = 8, stage 2 = 8, stage 3 = 6, stage 4 = 4 and stage 5= 4.

Supplementary Table 12. List of qPCR primers for *FMNL2* functional characterization

Gene	Forward Primer	Reverse Primer
House Keeping Genes		
ACTB	5' CCCTGAGGAGCACCCTGTGC 3'	5' GGCTGGGGTGTTGAAGGTCT 3'
MAPK1	5' TTGAACAGGCTCTGGCCCAC 3'	5' TGAATGGCGCTTCAGCAATGG 3'
Genes of Interest		
FMNL2	5' AACTCAGGATTAGCAGCTGTG 3'	5' ATCTGATTGGGCTTCAGGGC 3'
FMNL3	5' GACGTGCTGGTGGATTACCT 3'	5' CCGGAGCTTGTCAAATGCAC 3'

REFERENCES

1 Williams, P. A. *et al.* Vitamin B3 modulates mitochondrial vulnerability and prevents glaucoma in aged mice. *Science* **355**, 756-760, doi:10.1126/science.aal0092 (2017).

2 Banda, Y. *et al.* Characterizing Race/Ethnicity and Genetic Ancestry for 100,000 Subjects in the Genetic Epidemiology Research on Adult Health and Aging (GERA) Cohort. *Genetics* **200**, 1285-1295, doi:10.1534/genetics.115.178616 (2015).

3 Bailey, J. N. *et al.* Genome-wide association analysis identifies TXNRD2, ATXN2 and FOXC1 as susceptibility loci for primary open-angle glaucoma. *Nat Genet* **48**, 189-194, doi:10.1038/ng.3482 (2016).

4 Wiggs, J. L. *et al.* Common variants at 9p21 and 8q22 are associated with increased susceptibility to optic nerve degeneration in glaucoma. *PLoS Genet* **8**, e1002654, doi:10.1371/journal.pgen.1002654 (2012).

5 Li, Z. *et al.* A common variant near TGFBR3 is associated with primary open angle glaucoma. *Hum Mol Genet* **24**, 3880-3892, doi:10.1093/hmg/ddv128 (2015).

6 Burdon, K. P. *et al.* Genome-wide association study identifies susceptibility loci for open angle glaucoma at TMCO1 and CDKN2B-AS1. *Nat Genet* **43**, 574-578, doi:10.1038/ng.824 (2011).

7 Osman, W., Low, S. K., Takahashi, A., Kubo, M. & Nakamura, Y. A genome-wide association study in the Japanese population confirms 9p21 and 14q23 as susceptibility loci for primary open angle glaucoma. *Hum Mol Genet* **21**, 2836-2842, doi:10.1093/hmg/dds103 (2012).

8 Gharahkhani, P. *et al.* Common variants near ABCA1, AFAP1 and GMDS confer risk of primary open-angle glaucoma. *Nat Genet* **46**, 1120-1125, doi:10.1038/ng.3079 (2014).

9 Springelkamp, H. *et al.* New insights into the genetics of primary open-angle glaucoma based on meta-analyses of intraocular pressure and optic disc characteristics. *Hum Mol Genet* **26**, 438-453, doi:10.1093/hmg/ddw399 (2017).

10 Thorleifsson, G. *et al.* Common variants near CAV1 and CAV2 are associated with primary open-angle glaucoma. *Nat Genet* **42**, 906-909, doi:10.1038/ng.661 (2010).

11 Hoffmann, T. J. *et al.* Genome-wide association and admixture analysis of glaucoma in the Women's Health Initiative. *Hum Mol Genet* **23**, 6634-6643, doi:10.1093/hmg/ddu364 (2014).

12 Springelkamp, H. *et al.* ARHGEF12 influences the risk of glaucoma by increasing intraocular pressure. *Hum Mol Genet* **24**, 2689-2699, doi:10.1093/hmg/ddv027 (2015).

13 Chen, Y. *et al.* Common variants near ABCA1 and in PMM2 are associated with primary open-angle glaucoma. *Nat Genet* **46**, 1115-1119, doi:10.1038/ng.3078 (2014).

14 Wagner, A. H. *et al.* Exon-level expression profiling of ocular tissues. *Exp Eye Res* **111**, 105-111, doi:10.1016/j.exer.2013.03.004 (2013).

15 Bowes Rickman, C. *et al.* Defining the human macula transcriptome and candidate retinal disease genes using EyeSAGE. *Invest Ophthalmol Vis Sci* **47**, 2305-2316, doi:10.1167/iovs.05-1437 (2006).

16 Liu, Y. *et al.* Serial analysis of gene expression (SAGE) in normal human trabecular meshwork. *Mol Vis* **17**, 885-893 (2011).

17 Howell, G. R. *et al.* Molecular clustering identifies complement and endothelin induction as early events in a mouse model of glaucoma. *J Clin Invest* **121**, 1429-1444, doi:10.1172/JCI44646 (2011).
18 Howell, G. R., Walton, D. O., King, B. L., Libby, R. T. & John, S. W. Datgan, a reusable software system for facile interrogation and visualization of complex transcription profiling data. *BMC Genomics* **12**, 429, doi:10.1186/1471-2164-12-429 (2011).
