# Supplementary material for: A multiethnic genome-wide association study of primary open-angle glaucoma identifies novel risk loci
Source: Nat Commun. 2018 Jun 11;9:2278. doi: 10.1038/s41467-018-04555-4 (PMC5995837; doi:10.1038/s41467-018-04555-4)
Supplement: Supplementary file 4 — Description of Additional Supplementary Files [file 41467_2018_4555_MOESM4_ESM.pdf]

Description of Additional Supplementary Files

File Name: Supplementary Data 1
Description: For each of the associated signals identified in either GERA or UKB, we computed each variant's ability to explain the observed signal within a 2 Mb window (±1.0 Mb with respect to the original lead SNP) and derived the smallest set of variants that included the causal variant with 95% probability (95% credible set). The 24 credible sets include a total of 1,098 variants in 28 annotated genes. Abbreviations: CHR, chromosome, SNP, single nucleotide polymorphism; BP, base pair; A_min, minor allele; A_maj, major allele.
